# Pancreatic Mesenchyme Regulates Epithelial Organogenesis throughout Development

**DOI:** 10.1371/journal.pbio.1001143

**Published:** 2011-09-06

**Authors:** Limor Landsman, Amar Nijagal, Theresa J. Whitchurch, Renee L. VanderLaan, Warren E. Zimmer, Tippi C. MacKenzie, Matthias Hebrok

**Affiliations:** 1 Diabetes Center, Department of Medicine, University of California, San Francisco, San Francisco, California, United States of America; 2 Eli and Edythe Broad Center of Regeneration Medicine and Stem Cell Research, Department of Surgery, University of California, San Francisco, San Francisco, California, United States of America; 3 Department of Systems Biology and Translational Medicine, Texas A&M Health Science Center, College Station, Texas, United States of America; University of Cambridge, United Kingdom

## Abstract

The developing pancreatic epithelium gives rise to all endocrine and exocrine cells of the mature organ. During organogenesis, the epithelial cells receive essential signals from the overlying mesenchyme. Previous studies, focusing on ex vivo tissue explants or complete knockout mice, have identified an important role for the mesenchyme in regulating the expansion of progenitor cells in the early pancreas epithelium. However, due to the lack of genetic tools directing expression specifically to the mesenchyme, the potential roles of this supporting tissue in vivo, especially in guiding later stages of pancreas organogenesis, have not been elucidated. We employed transgenic tools and fetal surgical techniques to ablate mesenchyme via Cre-mediated mesenchymal expression of Diphtheria Toxin (DT) at the onset of pancreas formation, and at later developmental stages via in utero injection of DT into transgenic mice expressing the Diphtheria Toxin receptor (DTR) in this tissue. Our results demonstrate that mesenchymal cells regulate pancreatic growth and branching at both early and late developmental stages by supporting proliferation of precursors and differentiated cells, respectively. Interestingly, while cell differentiation was not affected, the expansion of both the endocrine and exocrine compartments was equally impaired. To further elucidate signals required for mesenchymal cell function, we eliminated β-catenin signaling and determined that it is a critical pathway in regulating mesenchyme survival and growth. Our study presents the first in vivo evidence that the embryonic mesenchyme provides critical signals to the epithelium throughout pancreas organogenesis. The findings are novel and relevant as they indicate a critical role for the mesenchyme during late expansion of endocrine and exocrine compartments. In addition, our results provide a molecular mechanism for mesenchymal expansion and survival by identifying β-catenin signaling as an essential mediator of this process. These results have implications for developing strategies to expand pancreas progenitors and β-cells for clinical transplantation.

## Abstract 2

Genetic disruption of the pancreatic mesenchyme reveals that it is critical for the expansion of epithelial progenitors and for the proliferation of insulin-producing beta cells.

## Author Summary

Embryonic development is a highly complex process that requires tight orchestration of cellular proliferation, differentiation, and migration as cells grow within loosely aggregated mesenchyme and more organized epithelial sheets to form organs and tissues. In addition to intrinsic cell-autonomous signals, these events are further regulated by environmental cues provided by neighboring cells. Prior work demonstrated a critical role for the surrounding mesenchyme in guiding epithelial growth during the early stages of pancreas development. However, it remained unclear whether the mesenchyme also guided the later stages of pancreas organogenesis when the functional exocrine and endocrine cells are formed. Here, we show that specific genetic ablation of the mesenchyme at distinct developmental stages in vivo results in the formation of a smaller, misshapen pancreas. Loss of the mesenchyme profoundly impairs the expansion of both endocrine and exocrine pancreatic progenitors, as well as the proliferative capacity of maturing cells, including insulin-producing beta-cells. Thus, our studies reveal unappreciated roles for the mesenchyme in guiding the formation of the epithelial pancreas throughout development. The results suggest that identifying the specific mesenchymal signals might help to optimize cell culture protocols that aim to achieve the differentiation of stem cells into insulin-producing beta cells.

## Introduction

Organogenesis is a complex and dynamic process that requires tight spatial and temporal regulation of differentiation, proliferation, and morphogenesis. The pancreas serves as an interesting model for the study of these processes as its epithelium gives rise to functionally distinct cells: endocrine cells, including insulin-producing β-cells that release hormones into the blood stream to regulate glucose homeostasis, and exocrine cells that produce, secrete, and transport digestive enzymes. These diverse cell types derive from common progenitors residing in the embryonic pancreatic epithelium through a well-orchestrated multi-step process. While numerous studies have delineated the cascades of transcription factors within the epithelium that guide epithelial cell development (reviewed in [1],[2]), the role of the surrounding mesenchyme in governing pancreas organogenesis at different stages remains largely unknown.

Mesenchymal cells start to coalesce around the nascent gut tube shortly before pancreas epithelial cells evaginate around mouse embryonic day 9.5 (e9.5) to form the dorsal and ventral buds [1]. At e13.5–e14.5 Pdx1^+^ epithelial precursor cells become committed to either the endocrine or the exocrine lineage, and from e15.5 until the end of gestation, pancreatic cells undergo final differentiation to give rise to all pancreatic cell types found in the adult organ. The first evidence that mesenchymal cells were required for pancreatic epithelial growth was provided in the 1960s by seminal work by Golosow and Grobstein [3], in which it was shown that e11 mouse pancreatic epithelium rudiments stripped of their overlying mesenchyme failed to grow in culture. However, further studies addressing the role of the mesenchyme at later stages have been difficult as the expanding pancreas epithelium quickly branches into the surrounding mesenchyme, thus preventing clean physical separation of these two layers after ∼e12 in the mouse. Additionally, while improved culture conditions for organ rudiments mimic embryonic development during early stages quite well [4], full replication of all in vivo aspects of later pancreas organogenesis have not been achieved ex vivo [5]. As a consequence, studying the role of the mesenchyme at advanced stages of pancreas development using explant systems resulted in controversial findings. A number of studies have shown that while mesenchymal cells have a positive effect on exocrine differentiation and growth in culture, they impair endocrine cell development [6]–[10]. Other studies have observed that close proximity between mesenchyme and epithelium promotes exocrine differentiation, while secreted mesenchymal factors enhance endocrine differentiation over a distance [5]. More recently, a study by Attali and colleagues showed that co-culture of epithelium with mesenchyme promotes the production of insulin-expressing cells, an effect largely due to the expansion of Pdx1^+^ precursor cells rather than maturation or proliferation of insulin-positive cells [11]. Importantly, endocrine development was highly variable and dependent on the culture conditions such as oxygen levels [11], further indicating that in vivo manipulation of mesenchymal gene expression is necessary to fully uncover all mesenchymal functions throughout pancreas development.

Starting in the 1970s, extensive efforts were made to identify mesenchymal factors responsible for these effects on the epithelial compartment [12],[13]. A decade ago, Bhushan and colleagues demonstrated that fibroblast growth factor 10 (Fgf10), expressed by mesenchymal cells from e9.5 until e11.5, is essential for pancreas growth and differentiation as it stimulates proliferation of Pdx1-expressing precursor cells [14]. Since then, germ-line knock out mouse lines, genetically manipulated zebra fish, and transfected chick embryos have been used to study a limited number of additional mesenchymal signaling pathways for their role in guiding pancreas formation (summarized in [1]). These studies provided evidence for Retinoic Acid (RA), Wnt, FGF, BMP, TGFβ, and EGF signaling pathways as important regulators of pancreas formation [1],[10],[14]–[17]. However, detailed studies of the requirement for individual mesenchymal factors in pancreas development have been hampered by the lack of transgenic tools that permit manipulation of gene expression specifically in the pancreatic mesenchyme.

Here, we present experiments that take advantage of *Nkx3.2* (*Bapx1*)-Cre transgenic mice in which Cre-expression is directed to the embryonic pancreatic mesenchyme, but not the epithelium. Using this Cre line in conjunction with mouse lines allowing Diphtheria Toxin (DT) induced apoptosis, we depleted mesenchymal cell during various stages of in vivo pancreas development. As expected, elimination of mesenchymal cells at the onset of pancreas development completely blocked pancreas organogenesis. Surprisingly, mesenchymal requirement was not restricted to this early stage, as ablation at later developmental stages also led to severe epithelial hypoplasia, reduced branching, and impaired β-cell and exocrine cell expansion. To elucidate the signaling pathways essential for mesenchyme function, we eliminated canonical Wnt signaling from the tissue. Loss of Wnt signaling within the mesenchyme resulted in mesenchymal cell ablation—subsequently leading to reduction in both exocrine and endocrine cell mass. Summarily, our results demonstrate that the pancreatic epithelium depends on mesenchymal signals for proper expansion and morphogenesis throughout development.

## Results

### *Nkx3*.2-Cre Directs Gene Expression to the Pancreatic Mesenchyme, but Not the Epithelium

In order to manipulate gene expression in pancreatic mesenchyme, but not epithelium, we looked for genes whose expression matches this pattern. Previous studies pointed to the homeobox gene *Nkx3.2* (also known as *Bapx1*), whose expression was found in the forming somites as well as in the mesenchyme of developing pancreas, stomach, and gut [18]–[23]. In contrast, *Nkx3.2* expression was not detected in endodermally derived cells in these tissues [18],[20],[23]. In the pancreatic mesenchyme *Nkx3.2* is expressed as early as e9.5, and by e12.5 its expression becomes restricted to the mesenchymal area, which will give rise to the splenic bud [18]–[20],[23]. An *Nkx3.2* (*Bapx1*)-Cre line, in which one copy of the endogenous *Nkx3*.*2* gene was replaced by a transgene encoding the Cre recombinase, had previously been generated [24],[25]. This transgenic mouse line faithfully replicates the endogenous expression of *Nkx3*.*2* and directs Cre activity to the foregut mesenchyme and skeletal somites starting at e9.5 [24].

**Figure 1:**
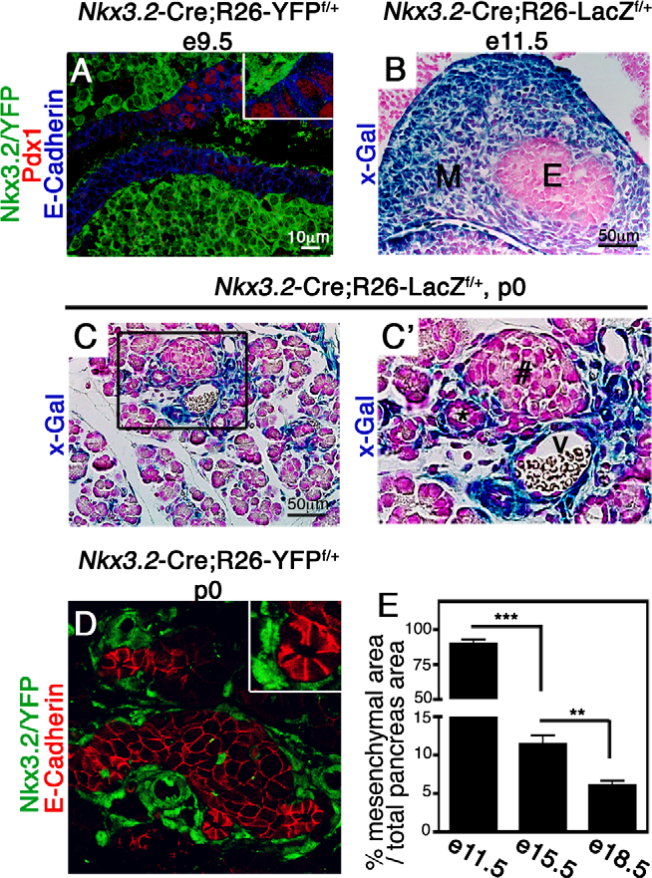
*Nkx3.2-Cre* drives gene expression in the embryonic pancreatic mesenchyme. (A) e9.5 *Nkx3.2*-Cre;R26-YFP^flox/+^ embryos were stained with antibodies against YFP (green), Pdx1 (red), and E-Cadherin (blue). YFP positive cells surround both the dorsal and ventral pancreatic epithelia and do not co-stain with the epithelial markers Pdx1 and E-Cadherin. Insert shows higher magnification of E-Cadherin^+^Pdx1^+^ and YFP^+^ cells (B,C,C′) *Nkx3.2*-Cre;R26-LacZ^f/+^ embryos stained with X-gal (blue) and counterstained with Fast Red (pink). (B) LacZ positive cells were found in the mesenchymal but not in the epithelial layer of the e11.5 pancreatic bud (B) and p0 pancreatic tissue (C, C′). (C′) A higher magnification of the areas marked with a box in (C). (D) p0 pancreatic tissues of *Nkx3.2*-Cre;R26-YFP^f/+^ stained for YFP (green) and E-Cadherin (red) to reveal clear separation between Nkx3.2/YFP^+^ cells and E-Cadherin^+^ epithelial cells. (E) Bar diagram shows the mesenchyme area as a percentage of total pancreatic area at the indicated days. *Nkx3.2*-Cre;R26-LacZ^f/+^ e11.5 pancreatic dorsal buds were stained as described in (B) and *Nkx3.2*-Cre;R26-YFP^f/+^ e15.5 and e18.5 pancreata were stained for YFP. The portion of Nkx3.2/YFP and Nkx3.2/LacZ areas were then measured as described in the Materials and Methods. *n* = 3. M, mesenchyme; E, endodermal epithelium; #, islet of Langerhans; *, duct; V, blood vessel. *p* values: **p<0.01, ***p<0.005.

Given that pancreatic expression of the *Nkx3.2*-Cre transgene was not thoroughly analyzed in prior studies, we first crossed the transgenic mice to two reporter strains, the R26-LacZ^f/+^ and the R26-YFP^f/+^ lines, which express LacZ or YFP, respectively, upon Cre-mediated recombination. YFP expression in *Nkx3.2*-Cre;R26-YFP^f/+^ embryos (from here on referred to as Nkx3.2/YFP) was not found in the endodermally-derived pancreatic epithelium marked by E-Cadherin and Pdx1 at e9.5 [26], but was ubiquitously detected in the surrounding mesenchyme (Figure 1A). Similarly, X-gal staining in *Nkx3.2*-Cre;R26-LacZ^f/+^ (from here on referred to as Nkx3.2/LacZ) indicated LacZ expression was confined to the surrounding mesenchyme at e11.5 (Figure 1B). At p0, Nkx3.2/LacZ and Nkx3.2/YFP expressing cells with fibroblast-like morphology were observed around islets, ducts, and blood vessels (Figure 1C,C′,D). Importantly, we could not detect reporter genes' expression in either epithelial (Figure 1C,C′,D), endothelial, or neuronal cells (Figure S1), indicating that *Nkx3.2*-Cre activity is excluded from those compartments throughout pancreatic development. Thus, the *Nkx3.2*-Cre line directs Cre-activity exclusively to the mesenchyme during pancreas development and serves as a novel tool to specifically manipulate embryonic gene expression in this tissue.

General histological analysis implied that the relative proportion of mesenchyme to epithelium shifts during pancreas organogenesis as epithelial cell numbers expand. We therefore took advantage of *Nkx3.2*-Cre transgenic mice to quantify the mesenchymal area during different developmental stages. By measuring the percentage of the pancreatic area marked by Nkx3.2/LacZ and Nkx3.2/YFP cells at various developmental stages, we determined that while the relative mesenchymal area is significantly reduced during pancreas organogenesis, it still comprised 11% and 6% of the pancreatic area at e15.5 and e18.5, respectively (Figure 1E). Thus, although there is a dramatic reduction in their portion over time, embryonic mesenchymal cells are present throughout pancreas organogenesis.

### Mesenchymal Cells Are Required to Support Early Stages of Pancreas Development In Vivo

**Figure 2:**
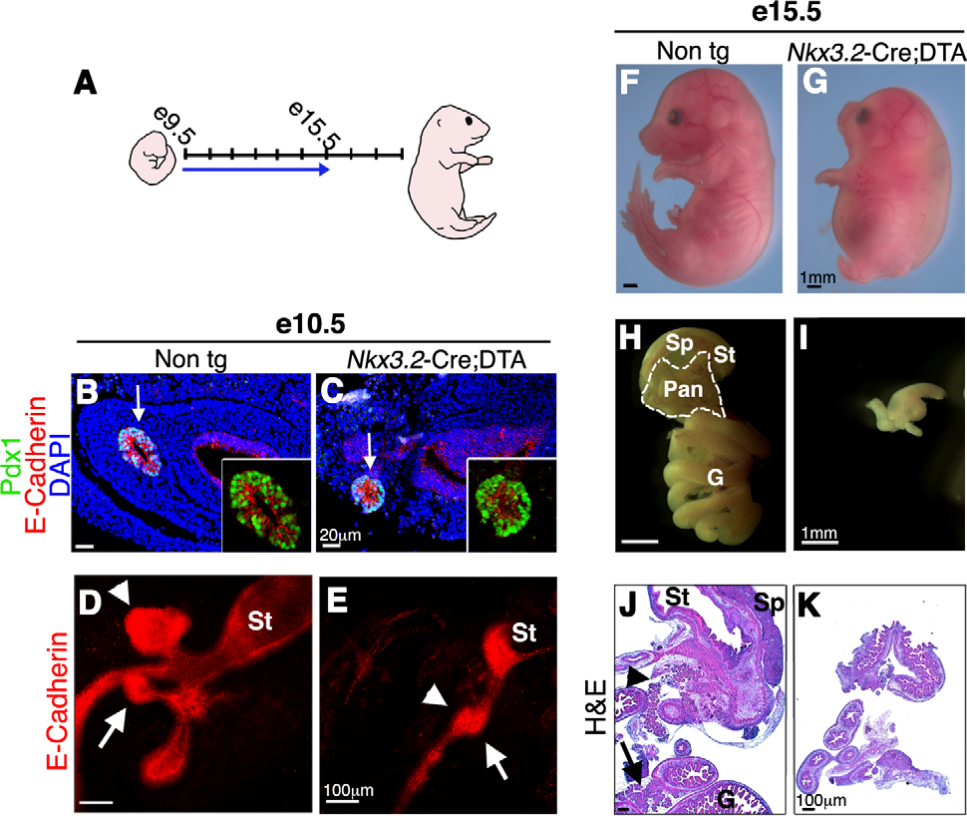
Depletion of pancreatic mesenchyme in *Nkx3.2-Cre;DTA* embryos inhibits epithelial growth. (A) A scheme illustrating embryonic development from e9.5 to e18.5. Arrow marks the time from the onset of mesenchymal Diphtheria Toxin A subunit (DTA) expression in pancreas to the time of analysis. (B–E) Analysis of e10.5 *Nkx3.2*-Cre;DTA embryos and non-transgenic littermates. (B,C) Pancreatic bud stained for Pdx1 (green), E-Cadherin (red), and DAPI (blue). The E-Cadherin^−^ mesenchymal layer completely surrounds Pdx1^+^E-Cadherin^+^ epithelial cells (marked with arrow) in control (B) but not in transgenic embryos (C). Inserts show higher magnification of the epithelial bud. (D,E) Whole mount staining against E-Cadherin marks pancreatic dorsal (arrowhead) and ventral (arrow) buds that are both smaller in transgenic embryos (E) as compared to control (D). St, Stomach. (F–K) Analysis of e15.5 *Nkx3.2-Cre;DTA* embryos and non-transgenic littermates. (F,G) Images show skeletal abnormalities in transgenic embryos (G) as compared to controls (F). (H–K) Gross morphology and histological analysis of embryonic gastrointestinal tract. (H,I) Isolated whole gastrointestinal tract. (J,K) Cross-sections stained with Hematoxylin and Eosin (H&E). Pancreatic tissue (Pan, outlined with a white dashed line in H and with arrows in J), stomach (St), spleen (Sp), and gut (G) are detected in control (H,J) but only indeterminate gut-like structures are found in transgenic (I, K) embryos.

Next, we tested the requirement for mesenchyme during pancreas organogenesis in vivo. Studies using cultured pancreatic rudiments as well as *Fgf10* knockout mice demonstrated a crucial role for the mesenchyme in expanding the pool of epithelial pancreatic precursor cells at early developmental stages (e9.5–e11.5) [3],[14],[27],[28]. In order to determine the role of the mesenchyme during pancreas development in vivo, we decided to ablate this tissue by employing transgenic mice carrying the Diphtheria Toxin (DT) active A subunit (DTA) flanked by flox sites (*R26-eGFP-DTA* mice [29], from here on referred to as DTA). Upon Cre-mediated recombination, the DTA produced by the transgene inhibits protein synthesis, resulting in rapid apoptosis of Cre-positive cells within less than 24 h [29]. Given that *Nkx3.2*-Cre is expressed in mesenchymal cells surrounding the pancreas from the time organ morphogenesis is initiated (e9.5, Figure 1A), *Nkx3.2-Cre;DTA* embryos permit the study of mesenchymal requirement at early stages of pancreas development (illustrated in Figure 2A).

We first analyzed potential defects in e10.5 embryos. At this stage, *Nkx3.2*-*Cre;DTA* embryos presented with Pdx1^+^E-Cadherin^+^ epithelial pancreatic cells (Figure 2B,C). However, while non-transgenic control pancreatic epithelial cells were completely surrounded by E-Cadherin^−^ mesenchymal cells, *Nkx3.2*-*Cre;DTA* embryos lacked most of the adjacent mesenchymal cell layer (Figure 2B,C). To assess potential defects in pancreatic bud morphology, we performed whole mount staining with the epithelial marker E-Cadherin. In wild-type embryos this staining revealed the expected organization of stomach, liver, and ventral and dorsal pancreatic buds (Figure 2D). In contrast, pancreatic buds of *Nkx3.2-Cre;DTA* embryos were severely reduced in size and did not evaginate from the foregut epithelium (Figure 2E).

*Nkx3.2-Cre;DTA* transgenic mice suffered from embryonic lethality starting at e15.5 as well as severe skeletal defects (Figure 2F,G) resulting from *Nkx3.2-Cre* activity in the somites [21],[22],[24]. Although the few viable *Nkx3.2-Cre;DTA* embryos recovered at e15.5 were only slightly smaller than non-transgenic littermates (Figure 2F,G), their gastrointestinal tract was dramatically reduced in size (Figure 2H,I), likely due to *Nkx3.2*-Cre-mediated expression of DTA in the mesenchyme surrounding these tissues [23],[24]. Notably, while pancreatic tissue was clearly detected in non-transgenic embryos at this stage (Figure 2H, demarcated by the white line), *Nkx3.2-Cre;DTA* embryos had no visible pancreatic tissue (Figure 2I). Histological analysis of gut rudiments confirmed the gross morphology observation and showed only intestine-like tissue in *Nkx3.2-Cre;DTA* embryos, with no discernable stomach, spleen, or pancreatic tissues (Figure 2J,K). Thus, elimination of mesenchyme at the earliest stages of pancreas formation leads to complete agenesis caused by the inability of pancreatic epithelium to evaginate from the forming gut and to expand.

### Mesenchymal Cell Ablation at Later Embryonic Stages Impairs Epithelial Pancreas Development

Pancreas development is a multistep process during which the epithelium undergoes complex morphological changes while common precursor cells differentiate into the various cells types that form the adult pancreas [30]. To test whether mesenchymal cells play distinct roles during different stages of pancreas development, we depleted the mesenchyme at various time points by injecting DT into developing embryos. Unlike primates, rodent cells lack a high affinity receptor for DT and therefore do not endocytose the toxin [31]. Since DT internalization into the cell cytoplasm is crucial for its ability to trigger the apoptotic machinery, rodent cells are resistant to ectopically administrated DT. However, mouse cells expressing a human DT Receptor (DTR) transgene, encoding for the human heparin binding epidermal growth factor (hbEGF), gain sensitivity to DT and are rapidly eliminated upon exposure to the toxin [32]. Prior studies have established that cell specific expression of human DTR in transgenic mice allows the ablation of targeted cells within 6 h following DT administration [33]. By crossing transgenic mice in which DTR expression is activated upon Cre-mediated recombination (iDTR [34], from here on referred to as DTR) with the *Nkx3.2*-Cre mice (*Nkx3.2-Cre;DTR*) we were able to specifically ablate the mesenchyme at different embryonic time points during pancreas development upon DT injection.

**Figure 3:**
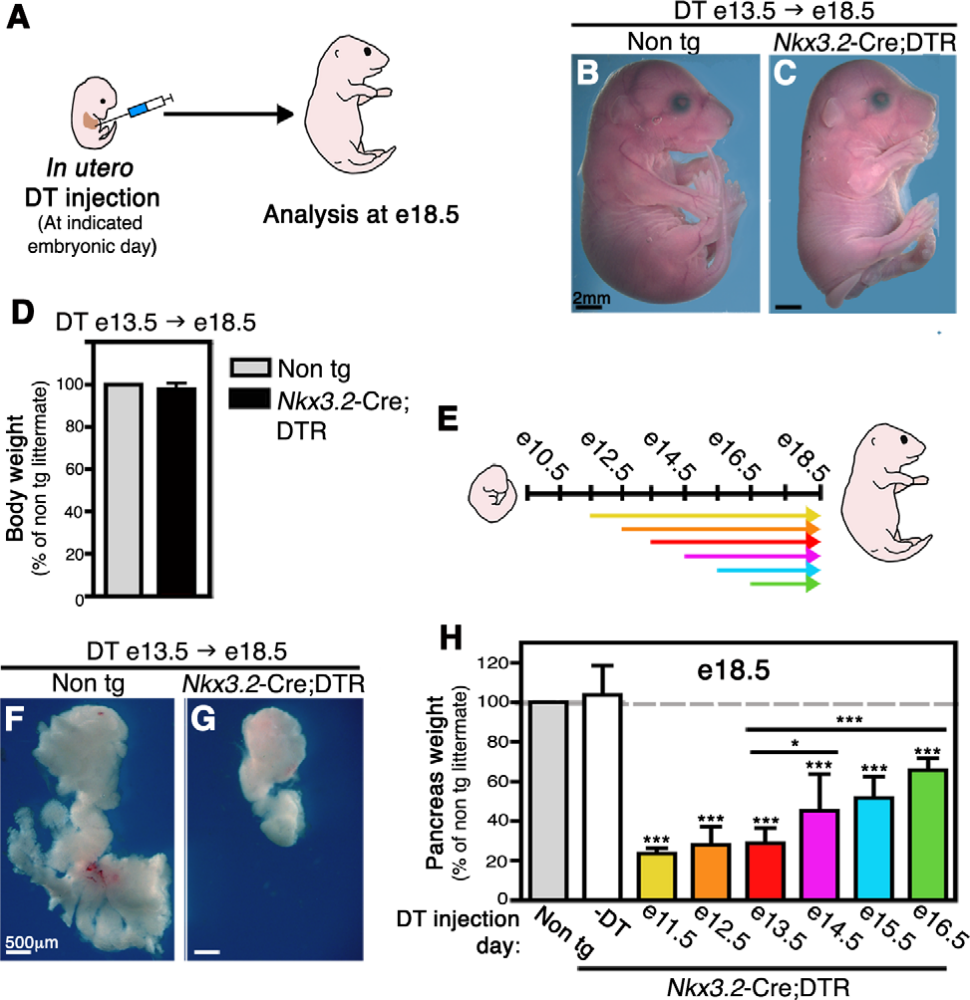
Pancreatic mesenchyme depletion at various developmental stages impairs organ development. *Nkx3.2-Cre;DTR* and non-transgenic littermates embryos were injected i.p. with a single dose of Diphtheria Toxin (DT) (8 ng/mg body weight) while in utero at indicated embryonic days. Embryos were then allowed to develop in situ until analyzed at e18.5, as illustrated in (A). (B–D) Embryos were injected with DT at e13.5 and analyzed at e18.5. (B,C) Whole body images reveal no gross defects in transgenic embryo (C) as compared to control littermate (B). (D) Body weight of transgenic (black bar) to non-transgenic (non tg, gray bar, set to 100%) littermates is equivalent. *n* = 5. (E) A scheme illustrating embryonic development from e9.5 to e18.5. Arrows mark the time between in utero DT injection to the day of analysis (e18.5). Different arrow colors represent different injection days. (F,G) Imaging of whole e18.5 pancreata, injected with DT at e13.5, reveals profound loss of pancreas tissue in DT transgenic embryos (G). (H) Bar diagram summarizing the relative pancreatic weight at e18.5 of *Nkx3.2*-*Cre;DTR* embryos either uninjected (-DT, empty bar) or DT-injected at e11.5 (yellow bar), e12.5 (orange bar), e13.5 (red bar), e14.5 (magenta bar), e15.5 (blue bar), or e16.5 (green bar). Non-transgenic littermates injected with DT at the corresponding days serve as controls (non tg, gray bar, set to 100%). Pancreas weight of uninjected transgenic embryos is comparable to control, whereas DT-injected transgenic pancreata weighed significantly less at all time points analyzed. *n*>5 for each group (from at least two independent litters). Student *t* test was used to compare the average weight of transgenic pancreata to non-transgenic littermates as well as to those injected with DT at e13.5 (indicated by horizontal lines). *p* value: *p<0.05, ***p<0.0001.

To ensure efficient delivery of DT to the developing pancreas, we injected the agent directly into embryos intraperitoneally (i.p.; the experimental procedure is illustrated in Figure 3A and Figure S2A–D) [35],[36]. As early as 4 h following DT injection into e13.5 *Nkx3.2*-Cre;*DTR* embryos, we observed an increase in apoptotic mesenchymal cells compared to controls (Figure S2E,F). One day after DT injection we detected only E-Cadherin expressing cells in *Nkx3.2-Cre;DTR* pancreata (Figure S2G,H), strongly indicating that E-Cadherin-negative mesenchymal cells were eliminated. The loss of *Nkx3*.2-Cre;*DTR*-positive mesenchymal cells was further confirmed by direct staining for human DTR expression (Figure S2I,J).

At the end of gestation (e18.5), *Nkx3.2-Cre;DTR* embryos injected with DT at e13.5 were viable and appeared grossly normal, with normal body weight (Figure 3B–D). At e18.5 the transgenic embryos displayed skeletal dysplasia, gastrointestinal defects, and asplenia (Figure 3B,C and Figure S3), likely a result of ablation of *Nkx3.2*-Cre expressing cells in these organs, and they died at birth. Therefore, in utero injections of DT into *Nkx3.2-Cre;DTR* do not cause embryonic lethality and permit studying the effects of mesenchyme ablation on epithelial pancreas development during embryogenesis.

To elucidate the requirement of mesenchyme at different stages we injected *Nkx3.2-Cre;DTR* embryos and non-transgenic littermates in utero with a single dose of DT at embryonic days 11.5, 12.5, 13.5, 14.5 ,15.5, or 16.5 (illustrated in Figure 3E). Embryos were then allowed to develop in situ until e18.5 when pancreata were dissected and weighed. Surprisingly, both dorsal and ventral pancreatic regions were significantly reduced in size in treated transgenic embryos independent of time of DT administration (Figure 3F–H). The most dramatic reduction in pancreas mass, up to 80%, was observed when transgenic embryos were injected between e11.5 and e13.5 (Figure 3H). DT injection at later stages, e14.5 and e15.5, resulted in an approximately 50% loss of pancreas mass. Notably, mesenchymal elimination as late as e16.5 led to a marked reduction in pancreas size to about two-thirds of non-transgenic littermates (Figure 3H). These results demonstrate that the mesenchyme is continuously required for proper pancreas development and organogenesis.

### Depletion of Mesenchymal Cells Affects Pancreas Morphology, but Not Pancreatic Cell Differentiation

Next, we performed an in-depth analysis of pancreas morphogenesis and cell differentiation in transgenic animals in which mesenchyme was depleted mid-way through organogenesis (DT injections into e13.5 *Nkx3.2-Cre;DTR* embryos followed by analysis at e18.5; DT e13.5→e18.5).

**Figure 4:**
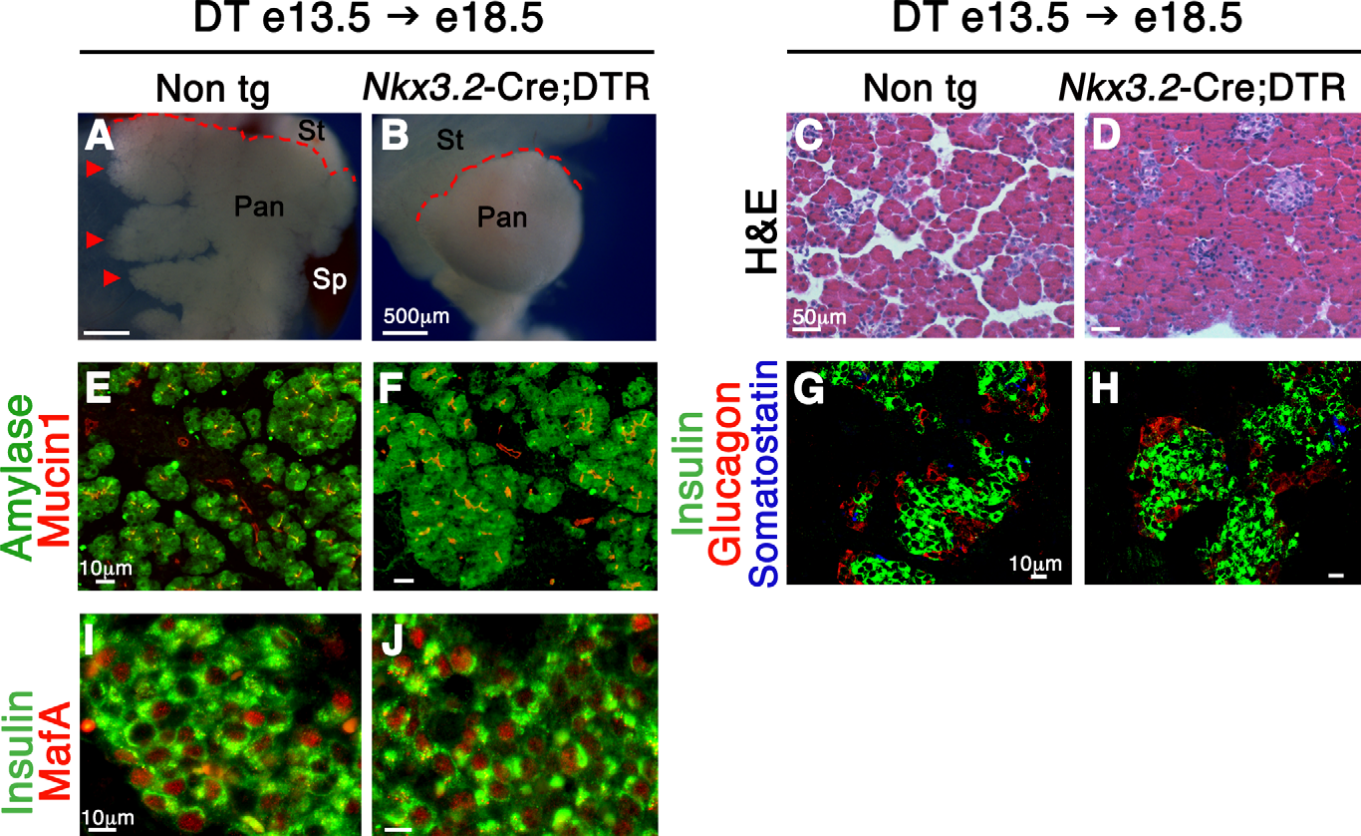
Depletion of pancreatic mesenchyme affects epithelial branching, but not cell differentiation. Morphological and histological analysis of *Nkx3.2-Cre;DTR* and non-transgenic littermates (non tg) in utero injected with DT at e13.5 and analyzed at e18.5. (A,B) Imaging of dorsal pancreata shows abnormal gross morphology of transgenic tissue (B). Typical left branches (arrowhead) found in non-transgenic pancreas are absent from the DT-treated transgenic tissue. In addition, DT-treated non-transgenic control present with an anvil-shaped tail, while transgenic pancreas have a rounded tail (red dashed lines). Pan, pancreas; St, stomach; Sp, spleen. (C,D) Histological analysis of pancreatic sections stained with H&E indicates abnormal and more condensed cellular distribution in transgenic pancreata (D), as compared to non-transgenic control (C). (E,F) Analysis for the acinar marker Amylase (green) and duct cell marker Mucin1 (red) reveals the presence of these two cell types in treated transgenic pancreata (F), similar to control (E). (G,H) Pancreatic sections were stained with antibodies against Insulin (green) as a β-cell marker, Glucagon (red) as a α-cell marker, and Somatostatin (blue) as δ-cell marker. Islet-like structures containing all these three endocrine cell types are found in the DT-treated transgenic embryos (H), similar to control (G). (I,J) Analysis for MafA (red) expression in β-cells (insulin^+^ cells, green) indicating normal cell maturation in transgenic pancreata (J).

When compared to normal tissues [37], mesenchyme-ablated pancreata displayed an abnormal globular morphology. DT-treated *Nkx3.2*-Cre;*DTR* pancreata were smooth and lacked the typical extension of the left branches (Figure 4A,B) as well as the gastric lobe (Figure 3F,G). In addition, DT-treated transgenic pancreata presented with a rounded tail instead of the stereotypical anvil-shaped tail found in non-transgenic controls (Figure 4A,B) [37]. Histological analysis further revealed more compacted cellular distribution in mesenchyme-ablated pancreata as compared to control, as shown by severe reduction of typical acellular areas normally found between adjacent lobes (Figure 4C,D).

Staining for the endothelial cell marker PECAM1 revealed that pancreatic vasculature, known to be crucial for organ development [38],[39], was not overtly disrupted in DT-treated transgenic pancreata (Figure S4A–D). Similarly, Tuj1 (β-III Tubulin)-expressing neuronal cells, known to be required for proper endocrine differentiation [40],[41], could be found in pancreata of DT-treated *Nkx3.2-Cre;DTR* embryos (Figure S4E,F).

Previous in vitro studies have implied that mesenchymal cells may control the differentiation of pancreatic epithelial cells [6],[9]. In order to study the in vivo effect of the mesenchyme on epithelial cell differentiation, we analyzed pancreatic tissues from DT-treated *Nkx3.2-Cre;DTR* for the expression of exocrine and endocrine markers at the end of gestation (DT e13.5→e18.5). Normal expression patterns for both the duct cell marker Mucin1^+^ and the acinar cell marker Amylase^+^ in treated transgenic pancreas indicated normal exocrine differentiation (Figure 4E,F). Furthermore, endocrine differentiation was not disturbed by mesenchymal ablation as Insulin, Glucagon, and Somatostatin expressing cells could be detected in transgenic pancreata (Figure 4G,H). Endocrine cells were single-hormone positive, clustered in islet-like structures typical for this developmental stage, and were distributed throughout the pancreas in a normal pattern (Figure 4C,D,G,H). Moreover, β-cells from transgenic embryos expressed the transcription factor MafA (Figure 4I,J), which is critical for full maturation and glucose responsiveness [42], strongly indicating that mesenchyme ablation does not block their differentiation potential. Thus, while pancreas morphogenesis is impaired upon mesenchyme elimination after the first stages of pancreas formation, differentiation of the major cell types was not blocked.

### Pancreatic Mesenchyme Is Required for Precursor Cell Proliferation

**Figure 5:**
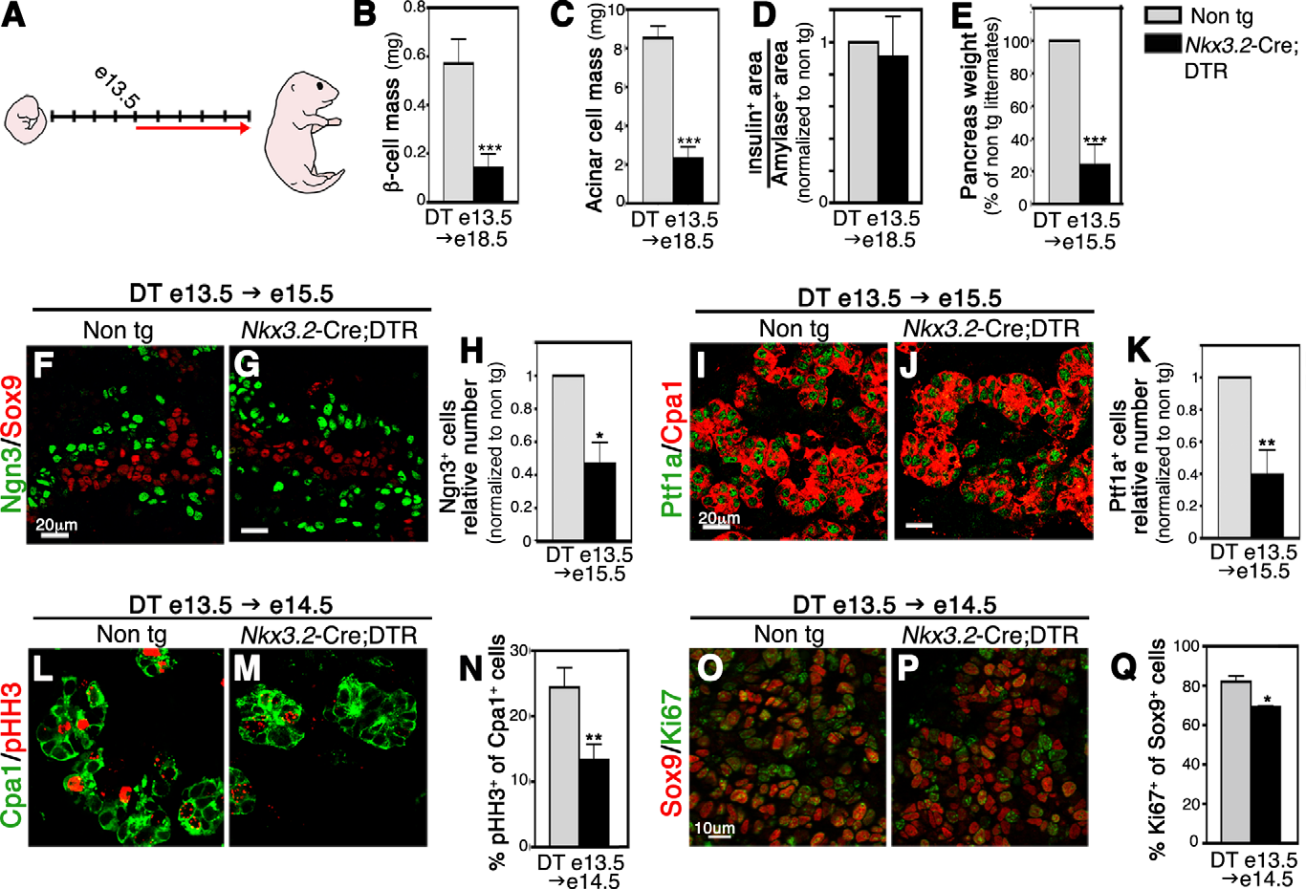
Mesenchymal ablation at e13.5 leads to reduced β- and acinar-cell mass due to impaired proliferation of precursor cells. *Nkx3.2*-Cre;DTR embryos and non-transgenic (non tg) littermates were injected with DT at e13.5 and analyzed at the embryonic days indicated. (A) A scheme illustrating embryonic development from e9.5 to e18.5. Red arrow marks the time from DT injection (e13.5) to analysis endpoint (e18.5). (B) Bar diagram shows marked reduction in β-cell mass in transgenic pancreata at e18.5 (black bar) compared to control tissue (gray bar). β-cell mass was calculated as the fraction of Insulin^+^ area out of the total pancreatic area multiplied by gross pancreatic mass. *n* = 3. (C) Analysis of acinar cells at e18.5 indicates a significant loss of acinar mass in transgenic pancreata (black bar). Acinar cell mass was calculated as described above for β-cell mass. *n* = 3. (D) Bar diagram depicting similar ratios between β (insulin^+^) and acinar (amylase^+^) -cell areas in transgenic samples (black bar) and control embryos (gray bar) at e18.5. Amylase^+^ and Insulin^+^ areas were calculated for each embryo (as described in the Material and Methods), and the Insulin^+^ area was divided by the Amylase^+^ area to obtain the relative ratio between the two components. For clarity, the ratios were normalized to those obtained from non-transgenic controls, which were set to “1.” *n* = 3. (E) Pancreatic weight of e15.5 embryos injected with DT at e13.5. Transgenic embryos (black bar) show reduced pancreatic weight in comparison to non-transgenic control littermates (gray bar, set to 100%). *n* = 4. (F–H) The number of Neurogenin 3 (Ngn3)-expressing cells is reduced in e15.5 transgenic pancreata. (F,G) Pancreatic tissues from DT injected non-transgenic (F) and *Nkx3.2-Cre;DTR* (G) embryos were stained for Ngn3 (green) and Sox9 (red), revealing normal expression pattern in the transgenic tissue at e15.5. (H) Ngn3-expressing cells were counted and their numbers were normalized to that found in non-transgenic pancreata. Number of Ngn3^+^ cells in *Nkx3.2-Cre;DTR* pancreata (black bar) was reduced by 50% when compared to non-transgenic littermates (non-tg, gray bar; total number of Ngn3^+^ cells was set to “1”). *n* = 3. (I–K) Reduced number of Ptf1a^+^ cells in transgenic pancreata. DT-treated non-transgenic (I) and transgenic (J) pancreata were stained for Ptf1a (green) and Cpa1 (red). (K) The number of Ptf1a^+^ cells in transgenic embryos (black bar) was reduced significantly by 40% when normalized to controls (non-tg, gray bar; total number of Ptf1a^+^ cells was set to “1”). *n* = 3. (L–N) Measurement of Cpa1^+^ cell proliferation demonstrates reduced rates in transgenic pancreata. e14.5 pancreatic tissues were stained against Cpa1 (green) and phosphorylated Histone H3 (pHH3, red) (L,M), and the percentage of Cpa1^+^pHH3^+^ cell as part of the Cpa1^+^ cell population was counted (N). Cpa1^+^ cells in transgenic pancreata (black bar) showed decreased proliferation as compared to non-transgenic control (non-tg, gray bar). *n* = 3. (O–Q) Reduced proliferation rate of Sox9^+^ precursor cells in transgenic embryos. e14.5 pancreatic tissues were stained against Sox9 (red) and Ki67 (green) (O,P). The percentage of proliferating Sox9^+^Ki67^+^ cells as part of the Sox9^+^ cell population (black bar) was reduced when compared to non-transgenic controls (non-tg, gray bar) (Q). *n* = 3. *p* value: *p<0.05, **p<0.01, ***p<0.005.

Although each of the specific pancreatic epithelium lineages formed in mesenchyme-depleted pancreata, the dramatic reduction in pancreatic organ size suggests a decrease in the overall number of pancreatic epithelial cells. In order to understand whether mesenchymal ablation affects either endocrine or exocrine mass, *Nkx3.2-Cre;DTR* mice treated with DT at e13.5 (illustrated in Figure 5A) were analyzed at e18.5 for β- and acinar cell masses. Both Insulin^+^ β-cell and Amylase^+^ acinar-cell mass were significantly reduced in transgenic mice when compared to non-transgenic littermates (Figure 5B,C), suggesting a requirement for mesenchymal cells during the expansion of both exocrine and endocrine compartments/precursors. The observation that transgenic pancreata maintained a normal acinar to β-cell ratio (Figure 5D) indicates that both cell types depend in equal measures on mesenchymal signals for their proliferation.

To determine the developmental stage during which mesenchyme ablation affects pancreatic mass, we injected embryos at e13.5 and investigated pancreata 2 d later at e15.5 (DT e13.5→e15.5). At that stage, pancreas mass in transgenic embryos was already reduced by 80% as compared to controls (Figure 5E), similar to the reduction observed in pancreata injected at e13.5 and analyzed at e18.5 (Figure 3H). Since mesenchymal cells comprise only 11% of pancreatic tissue at e15.5 (Figure 1E), the observed reduction in pancreatic weight was likely due to a rapid and significant loss of the epithelial compartment of the organ.

Next, we investigated whether cells of either the endocrine or exocrine compartments were already affected in e15.5 *Nkx3.2*-Cre;DTR embryos that were DT-treated 2 d before (i.e., at e13.5). While present in DT-treated transgenic pancreata (Figure 5F,G), the number of cells positive for Neurogenin 3 (Ngn3), a transcription factor that marks endocrine precursor cells [43], was significantly reduced compared to littermate control mice (Figure 5H). Similarly, the number of cells expressing Ptf1a, a transcription factor found in exocrine precursor and differentiated acinar cells [44], was significantly reduced 2-fold in DT e13.5→e15.5 *Nkx3.2-Cre;DTR* pancreata as compared to non-transgenic controls (Figure 5I–K). Therefore, the reduction in β-cell and acinar cell mass detected at DT e13.5→e18.5 *Nkx3.2-Cre;DTR* embryos (Figure 5B,C) is, at least in part, due to the decreased number of Ngn3^+^ precursor cells and Ptf1a^+^ exocrine cells at earlier developmental stages.

Since pancreatic growth between e13.5 and e15.5 relies heavily on proliferation of precursor cells [45], we next analyzed the effect of mesenchymal depletion on the proliferation rate of these cell populations in e14.5 *Nkx3.2-Cre;DTR* embryos treated at e13.5 (DT e13.5→e14.5). Epithelial tip cells serve as multi-potent progenitors before they become committed to the exocrine lineage around e14.5 [44]. Staining these cells, identified as Carboxypeptidase 1 (Cpa1) expressing cells, with an antibody against phosphorylated Histone H3, a marker of cell proliferation, revealed a 50% reduction in proliferating tip cells in *Nkx3.2-Cre;DTR* embryos compared to controls (Figure 5L–N). In addition, we analyzed proliferation of Sox9 expressing cells, a transcription factor that marks epithelial precursor cells giving rise to exocrine cells as well as to Ngn3^+^ endocrine precursors [46]–[48]. The percentage of Sox9^+^ proliferating cells was slightly but significantly smaller in transgenic embryos (Figure 5O–Q). We could not detect apoptotic epithelial cells by TUNEL (terminal deoxynucleotidyl transferase dUTP biotin nick end labeling) assays (unpublished data), concluding that depletion of mesenchymal cells affects both endocrine and exocrine mass through reduced proliferative capacity of epithelial progenitor cells rather than their apoptosis.

### Mesenchymal Cells Are Required for Proliferation of Differentiated Epithelial Cells

**Figure 6:**
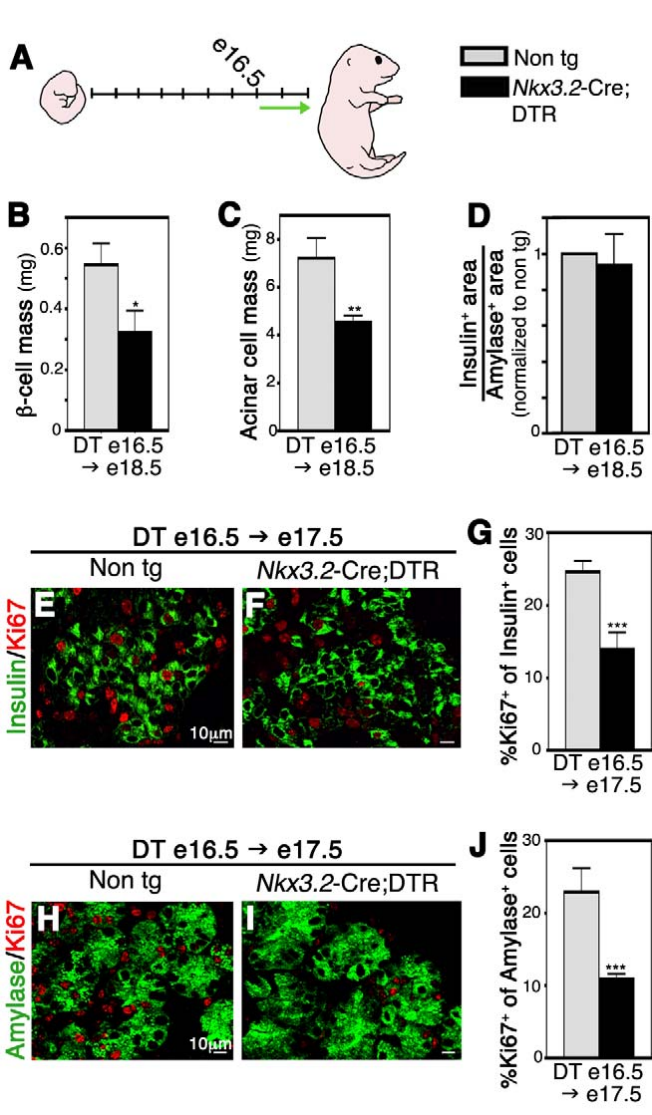
Mesenchymal depletion toward the end of gestation impairs acinar and β-cell proliferation. *Nkx3.2*-Cre;DTR and non-transgenic littermates were injected with DT in utero at e16.5 and analyzed at indicated embryonic days. (A) A scheme illustrating embryonic development from e9.5 to e18.5. Green arrow marks the time from DT injection (e16.5) to analysis endpoint (e18.5). (B) Analysis for β-cell mass (as described for Figure 5) at e18.5 shows significant reduction in transgenic pancreata (black bar) compared to controls (non-tg, gray bar). *n* = 3. (C) Bar diagrams show reduced acinar cell (as described for Figure 5) mass at e18.5 in transgenic pancreata (black bar) compared to non-transgenic littermates (non-tg, gray bar). *n* = 3. (D) β- to acinar-cell ratio is similar in transgenic and non-transgenic controls. Bar diagram presents the normalized ratio between β- and acinar-cell area at e18.5, as described for Figure 5. *n* = 3. (E–G) Reduced β-cell proliferation in transgenic pancreata (F,G, black bars) as compared to controls (E,G, gray bars). E17.5 pancreatic tissues were stained against Insulin (green) and Ki67 (red), and the percentage of double positive cell within the Insulin^+^ cell population was counted. *n* = 3. (H–J) Reduced Acinar cell proliferation in transgenic embryos (I,J, black bars) compared to control embryos (H,J, gray bars). (H,I) e17.5 pancreatic tissues stained against Amylase (green) and Ki67 (red). The percentage of Amylase^+^Ki67^+^ cell as part of the Amylase^+^ cell population was counted (J). *n* = 3. *p* value: *p<0.05, **p<0.01, ***p<0.005.

The reduced proliferative potential of progenitor cells at e14.5 explained, at least in part, the reduction in pancreas mass in embryos treated with DT at e13.5. However, when mesenchyme was eliminated at e16.5 we also observed a significant reduction of about 35% in pancreas mass at e18.5, affecting both the endocrine and exocrine compartments (DT e16.5→e18.5; Figures 3H, 6A–D). By e16.5, the various pancreatic cell types are committed towards their final differentiation fate and present with many of their mature cell characteristics. Since pancreatic growth at those late stages of development is attributed to proliferation of these differentiated cells [49], the decrease in pancreatic mass could not be due to reduced proliferation of progenitor cells. While previous studies did not detect effects of mesenchymal cells on β-cell proliferation in culture [11], in vivo analysis of *Nkx3.2-Cre;DTR* embryos treated with DT at e16.5 and analyzed at e17.5 revealed decreased proliferative potential of both insulin and amylase expressing cells (Figure 6E–J). In agreement with what we had found at earlier stages, the ratio between Insulin^+^/Amylase^+^ areas was not affected in the DT-treated embryos (Figure 6D), suggesting mesenchymal factors have similar effect on cells of these two compartments.

### Mesenchymal Wnt Signaling Is Required for Epithelial Growth by Maintaining the Mesenchymal Layer

Upon determining the requirement for mesenchymal cells to guide epithelial organ formation throughout development, we set out to identify signals and pathways critical for the mesenchymal effects. Canonical Wnt signaling is active in the developing pancreas, and both the mesenchyme and the epithelium express various Wnt ligands and receptors in a dynamic fashion [50]. At e11.5, Wnt signaling is observed in epithelial cells, and its level of activation declines in the following embryonic days [51], while its activity in the mesenchymal layer has been first reported around e13.5 [15],[52].

In order to directly investigate the role of mesenchymal Wnt signaling in pancreas development, we decided to block this pathway specifically in the mesenchyme by crossing transgenic mice carrying floxed alleles of β-catenin (βcat^f/f^), an essential mediator of canonical Wnt signaling, with *Nkx3*.2*-Cre* mice. In addition to its critical role in Wnt signaling, β-catenin has other functions within cells, most notably in maintaining cell-cell interactions as part of a complex with E-Cadherin. However, in pancreatic mesenchymal cells we failed to observe membrane-associated localization of the β-catenin protein (Figure S5A). Therefore, elimination of this gene in *Nkx3.2*-Cre;*β-cat^f/f^* pancreata is unlikely to perturb cell-cell interactions but should reveal the requirement for β-catenin mediated Wnt signaling in mesenchyme.

**Figure 7:**
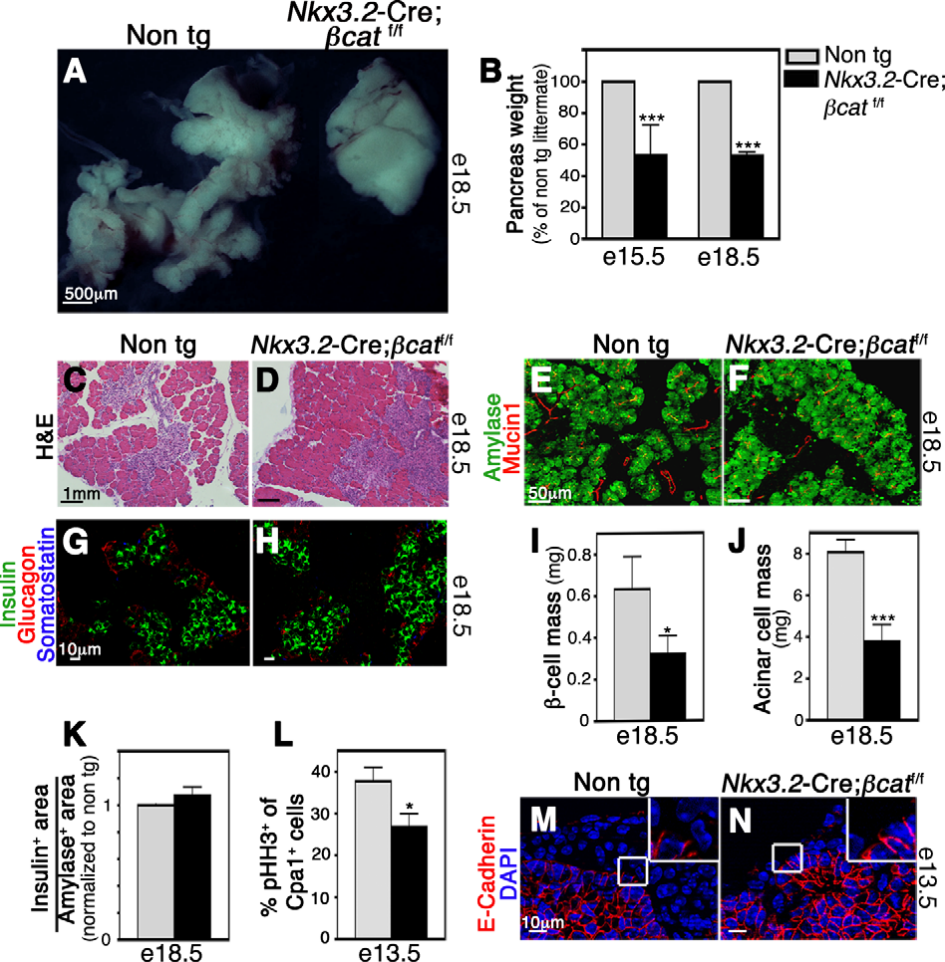
Elimination of mesenchymal Wnt signaling impairs pancreas formation. Pancreatic tissues from *Nkx3.2*-Cre;*βcat* ^f/f^ and non-transgenic (non-tg) littermates were analyzed at indicated time points. (A) Gross morphology shows smaller transgenic pancreas (right) with aberrant branching morphology. (B) Reduced pancreatic mass in e15.5 and e18.5 mutant embryos (black bars) as compared to non-transgenic littermates (non-tg, gray bars). For clarity, pancreatic mass in control mice was set to 100%. *n* = 5. (C,D) Histological analysis of pancreatic sections stained with H&E reveals abnormal tissue morphology in e18.5 mutant embryos (D) when compared to control (C). (E,F) Amylase^+^ (green) acinar cells and Mucin1^+^ (red) duct cells can be found in mutant pancreata at e18.5 (F). (G,H) Endocrine cells are present and express mature markers in e18.5 mutant pancreata (H), similar to control (G). Tissues were stained with antibodies against Insulin (green), Glucagon (red), and Somatostatin (blue). (I) Reduced β-cell mass (quantification described in Figure 5) in e18.5 mutant pancreata (black bar) as compared to control (gray bar). *n* = 3. (J) Reduced Acinar cell mass (quantification described in Figure 5) in mutant pancreata at e18.5 (black bar) as compared to non-transgenic littermates (gray bar). *n* = 3. (K) The ratio between β- and acinar cell areas (as described in Figure 5) at e18.5 is maintained in mutant pancreata (black bar) when compared to non-transgenic littermates (gray bar, set to “1”). *n* = 3. (L) Decreased proliferation of Cpa1^+^ precursor cells in mutant pancreata at e13.5. Bar diagram shows the percentage of pHH3^+^Cpa1^+^ cells out of the whole Cpa1^+^ population in mutants (black bar) and non-transgenic pancreata (gray bar). *n* = 3. (M,N) e13.5 pancreatic tissue stained with E-Cadherin (red) and counter-stained with DAPI (blue) shows absence of E-Cadherin^−^ mesenchymal cells in mutant tissues (N). Inserts represent higher magnification of the areas marked with white frames. *p* values: *p<0.05, **p<0.01, ***p<0.005.

As expected, elimination of β-catenin did not affect epithelial size at e12.5 (Figure S5B) prior to the reported onset of mesenchymal Wnt signaling. In contrast, *Nkx3.2-Cre;β-cat^f/f^* pancreata were markedly reduced in size at e15.5 and e18.5 (Figure 7A,B), indicating that mesenchymal β-catenin signaling is critical for organ formation at later stages. In addition, *Nkx3.2-Cre;βcat^f/f^* pancreata exhibited aberrant morphology with diminished branching when compared to controls (Figure 7A,C,D).

In order to identify the potential effects on pancreatic epithelial development in *Nkx3.2-Cre;βcat^f/f^* embryos, we stained e18.5 knock-out pancreata for various cell markers and assessed acinar- and β-cell mass. All major pancreatic cell types, both of the exocrine (acinar and duct cells, Figure 7E,F) and of the endocrine compartments (α-, β-, and δ-cells, Figure 7G,H), were detected in the *Nkx3.2-Cre;βcat^f/f^* e18.5 pancreata. However, both β-cell and acinar-cell mass was significantly reduced in knock-out embryos (Figure 7I,J). Interestingly, the ratio between β- and acinar cells was maintained in *Nkx3.2-Cre;βcat^f/^* ^f^ pancreata (Figure 7K).

The Wnt signaling pathway was shown to become activated in the pancreatic mesenchyme around e13.5 [15],[52]. To address whether the reduction in pancreatic mass observed in *Nkx3.2-Cre;βcat^f/f^* at e15.5 and e18.5 is due to effects on epithelial growth at earlier stages, we studied epithelial proliferation in these mice at e13.5. At this stage, proliferating Cpa1^+^ tips cells serve as multipotent pancreatic progenitor cells for both endocrine and exocrine populations [44]. As shown in Figure 7L, the proliferation rate of Cpa1^+^ cells was significantly lower in *Nkx3.2-Cre;βcat^f/f^* embryos as compared to controls. Cell death was not apparent as we could not detect apoptotic epithelial cells by TUNEL assays or by staining for cleaved Caspase3 (unpublished data). Therefore, blocking mesenchymal Wnt signaling leads to reduced pancreatic mass by affecting the proliferation capacity of epithelial precursor cells.

Wnt signaling is known to regulate cell survival and proliferation [53]. Since pancreata from *Nkx3.2-Cre;βcat^f/f^* mice phenocopied those from DT-treated *Nkx3.2-Cre;DTR* mice, we wondered whether Wnt signaling is required for mesenchymal cell survival. Indeed, while e13.5 pancreatic tissue from wild type embryos contained both E-Cadherin-positive epithelial cells and E-Cadherin-negative mesenchymal cells (Figure 7M), we could detect only E-Cadherin expressing cells in *Nkx3.2*-Cre;βcat^f/f^ tissues (Figure 7N), indicating ablation of the pancreatic mesenchymal layer in transgenic mice. Thus, our results point to mesenchymal Wnt signaling as a critical mediator of mesenchymal cell survival in vivo and therefore of epithelial growth and patterning.

## Discussion

Despite extensive efforts, the role of the mesenchyme during in vivo pancreas development has remained elusive. Due to the absence of suitable genetic tools, probing the role of the pancreatic mesenchyme during organogenesis has been mainly restricted to organ rudiment culture experiments. In addition to the inability to faithfully mimic the in vivo conditions, clean separation of mesenchyme and epithelium for culture experiments is limited to early stages of pancreas organogenesis before the epithelial layer has integrated into the overlying mesenchyme (prior to e12.5 in the mouse). Here, we show that *Nkx3.2*-Cre mice permit transgene manipulation specifically in mesenchyme, but not in other pancreatic tissues. By using this tool to eliminate pancreatic mesenchymal cells at will, we expand upon classical tissue culture studies and for the first time present a model system suitable for detailed analysis of the various functions of this supporting tissue in vivo at multiple gestational ages.

A key finding of our studies is the observation that pancreatic mesenchyme provides critical functions for the proper development of the epithelial compartment throughout organogenesis. As expected from previous studies [3],[14], depletion of mesenchymal cells at the onset of pancreas development using *Nkx3*.2-*Cre;DTA* mice arrests pancreas organogenesis. Mesenchymal ablation at later stages, by injecting DT into *Nkx3.2-Cre;DTR* transgenic embryos at various stages or by blocking mesenchymal Wnt signaling in *Nkx3.2-Cre;βcat^f/f^* mice, impairs pancreatic epithelium growth and branching. Notably, while the morphological changes observed are profound, we did not observe alterations in cell differentiation capacity of the three main pancreatic cell types, the endocrine, acinar, and duct cells. However, our results clearly demonstrate a requirement of the mesenchyme for the expansion of epithelial progenitor cells, as well as proliferation of differentiated pancreatic cells.

### Requirement of Secreted Mesenchymal Factors During Pancreas Development

Factors secreted by the pancreas mesenchyme have previously been shown to regulate pancreas organogenesis [6], including Fgf10 whose function is required for the expansion of common epithelial progenitor cells during early stages of pancreas development [14]. The pancreatic defects we observe in *Nkx3.2*-Cre;*DTA* embryos are more severe than those previously reported for *Fgf10* ^−/−^ pancreata [14], a finding likely explained by the absence of mesenchymal cells, and thus reduction of all mesenchymal factors, in transgenic mice.

Our results further demonstrate a requirement for mesenchymal cells in promoting proliferation of various epithelial cell types, including precursors and differentiated cells. While it is theoretically possible that these functions are mediated by a limited number of factors throughout all stages of development, the dynamic activation of mesenchymal signaling pathways (summarized in [1]) would suggest a more complex interplay of a diverse set of molecules that changes over time. Our findings also suggest that mesenchyme supports proliferation of multiple distinct cell types, even during the same developmental stage. For instance, mesenchyme ablation has similar effects on proliferation of mature acinar and β-cells towards the end of gestation. This observation poses the question as to whether different epithelial cell types rely on the same mesenchymal factor(s) for their proliferation, or whether these processes are mediated by distinct signals. Future analysis is required to identify secreted factors expressed by the pancreatic mesenchyme at different developmental stages. The use of the *Nkx3.2*-Cre line will allow specific manipulation of the genes coding for these signals to ascertain their role during pancreas organogenesis.

### Mesenchyme Governs Endocrine and Exocrine Development and Growth

Another important finding concerns the observation that the pancreatic mesenchyme is required for both endocrine and exocrine development in vivo. Previous reports had reached differing conclusions, with some demonstrating a positive role for the mesenchyme on exocrine formation but not endocrine cell development [6],[7],[9], and others indicating that mesenchymal factors promote proliferation of multi-potent pancreas progenitors that subsequently increase the formation of endocrine cells [11]. Some of these conflicting results can be explained by the different culture conditions used in each experiment.

In contrast to the cultured studies, in vivo depletion of the mesenchyme investigated here revealed similar requirements for this tissue with regard to the endocrine and exocrine cytodifferentiation. At this point, we cannot exclude that other cells types, including endothelial and neural-crest derived cells [38]–[41], or cells residing in the adjacent liver, stomach, gut, or kidneys might provide signals that guide epithelial cell differentiation in mesenchyme depleted embryos in vivo. In addition, mesenchymal cells that did not originate from *Nkx3.2*-Cre expressing cells might still be present in our in vivo model and could provide either instructive or permissive signals.

Prior organ culture studies proposed another model to explain the various effects of the mesenchyme on the epithelial compartments by demonstrating distinct effects of the mesenchyme on epithelial cells depending on the physical distance and contact between these tissues [5]. In these experiments, close proximity between epithelial and mesenchymal cells promoted exocrine differentiation while at the same time blocked endocrine formation. In contrast, mesenchyme factors supported endocrine differentiation at a distance, indicating that the physical relation between mesenchymal and epithelial cells is critical for endocrine versus exocrine differentiation. Our studies support the notion of mesenchymal signals being important for both endocrine and exocrine development. However, our lineage tracing experiments provide evidence of close physical contact between Nkx3.2/LacZ and Nkx3.2/YFP expressing cells with endocrine cells, indicating that close proximity between mesenchymal and epithelial cells does not necessarily interfere with endocrine differentiation. However, since mesenchymal cells surround islets, they are likely in close contact only with peripheral endocrine cells, such as α-cells, while direct interactions with centrally located β-cells might not be common. Whether the mesenchyme contributes to β-cell expansion by releasing secreted factors or through cell-cell interactions as well as how the mesenchyme affects other endocrine cells are questions that need to be addressed in future experiments. Furthermore, isolation and characterization of mesenchymal cells throughout development might reveal cell heterogeneity that could explain differential functions with regard to promoting endocrine versus exocrine development.

### Requirement of Mesenchymal Signaling Pathways in Pancreas Organogenesis

Our results also point to sustained mesenchyme function as a critical regulator of epithelial pancreas development and identify Wnt signaling as an essential mediator of mesenchyme survival. It is not clear as to whether Wnt signaling is activated in an autocrine or paracrine manner, as several Wnt ligands are expressed by both pancreatic epithelial and mesenchymal cells during development [50]. It is noteworthy that the defects we observe in *Nkx3.2*-Cre;*βcat* ^f/f^ only occur after the onset of canonical Wnt signaling in pancreas mesenchyme as measured by expression of transgenic Wnt-reporters (i.e., e13.5 [15],[52]). The implication of canonical Wnt signaling as the cause for the observed phenotypes is indirectly supported by a previous study using germ-line knock-out mice in which *mPygo2*, a critical component of the nuclear β–catenin/Tcf complex required for β-catenin transcriptional activity, has been eliminated [15]. *mPygo* ^−/−^ mice show pancreas hypoplasia and a reduction in endocrine mass [15], phenotypes that are not observed when this gene is specifically eliminated in pancreas epithelium. Thus, while mesenchyme specific depletion of *mPygo2* has not been reported, the absence of pancreas hypoplasia upon epithelial-specific *mPygo2* elimination suggests that at least some of the pancreatic defects are caused by reduced mesenchymal Wnt signaling. However, and in contrast to *Nkx3.2*-Cre;*βcat* ^f/f^ pancreata, the exocrine compartment is not affected in *mPygo2* ^−/−^ mutants and mesenchyme depletion was not reported in those mice. Since Wnt signaling is significantly reduced, but not completely blocked in the absence of *mPygo2* [15], it is possible that low level of canonical Wnt signaling is sufficient for mesenchymal cell survival and the production of factors that promote exocrine cell development. Alternatively, β-catenin is known to regulate cell-cell interactions as part of Cadherin complexes and these additional functions might be crucial for the maintenance of the pancreatic mesenchyme. However, we did not observe β-catenin localized to membranes in mesenchymal cells. In order to study whether different levels of mesenchymal Wnt signaling have a different effect on endocrine and exocrine expansion, mice specifically lacking mesenchymal expression of various components of this pathway (such as mPygo2) would need to be examined.

In addition to Wnt signaling, other signaling pathways, such as the RA, BMP, and Hedgehog, have been implicated as mesenchymal factors regulating pancreas development [15]–[17],[54],[55]. Using *Nkx3*.2-Cre line as a novel tool to manipulate gene expression in the pancreatic mesenchyme will allow direct study of the role of these and potentially other pathways in pancreas organogenesis.

In summary, data presented here indicate continuous requirement of mesenchymal cells and/or mesenchyme-derived signals to regulate epithelial pancreas formation from the onset of organ morphogenesis until the end of gestation. Isolation of mesenchymal cells at different stages of pancreas formation might allow identification of candidate factors that regulate expansion of common and endocrine progenitors as well as of differentiated β-cells. Future therapies for both type I and II diabetes rely on renewable sources of functional insulin-producing β-cells [56]. Current protocols allow the formation of pancreas progenitor cells from human embryonic stem cells (hESC) in vitro, but not fully differentiated β-cells. Our results demonstrate that mesenchymal factors provide critical signals for the expansion of both precursors and differentiated endocrine and exocrine cells. Thus, mesenchymal signaling factors not yet identified will likely be useful for expansion of hESC derived pancreas progenitor and differentiated β-cells.

## Material and Methods

### Mice

Mice used in this study were maintained according to protocols approved by the Committee on Animal Research at the University of California, San Francisco. *Nkx3.2 (Bapx1)-Cre* mice were described previously [24]. *R26*-*YFP^flox^* (*Gt(ROSA)26Sor^tm1(EYFP)Cos^*), *R26-LacZ^flox^* (Gt(ROSA)26Sor^tm1Sor^), *R26-eGFP-DTA* (*Gt(ROSA)26Sor^tm1(DTA)Jpmb^*), *DTR* (iDTR, *Gt(ROSA)26Sor^tm1(HBEGF)Awai^*), and *β-catenin^flox^* (*Ctnnb1^tm2Kem^*) mice were obtained from Jackson Laboratories. Noon on the day a vaginal plug was detected was considered as embryonic day 0.5.

### Diphtheria Toxin (DT) Injections

Injections were preformed as previously described [35],[36]. Briefly, pregnant females were anesthetized, a laparotomy was performed, and the uterus was delivered through the incision (as illustrated in Figure S2A–D). Each embryo was micro-injected with 8 ng/gr body weight Diphtheria Toxin (Sigma) diluted in 5 µl PBS. The uterus was placed back into the abdominal cavity and the laparotomy was closed. Embryos were allowed to develop in situ until indicated stages.

### Immunohistochemistry

For immunofluorescence, dissected embryos and pancreatic tissues were fixed with Z-fix (Anatech) for 2–16 h, embedded in paraffin wax, and sectioned. For Ptf1a staining, tissues were fixed with Z-fix for 2 h, embedded in OCT (Tissue Tek), and cryosectioned. Tissue sections were stained using the following primary antibodies: rabbit anti-Amylase (1∶200, Sigma), goat anti-Cpa1 (1∶200, R&D), mouse anti-E-Cadherin (1∶200, BD), rabbit anti-Glucagon (1∶200, Linco), guinea pig anti-Insulin (1∶200, Linco), mouse anti-Ki67 (1∶200, BD), rabbit anti-MafA (1∶200, Bethyl), armenian hamster anti-Mucin1 (1∶200, Neomarker), guinea pig anti-Neurogenin 3 (1∶400, Millipore), rabbit anti-phosphorylated Histone H3 (1∶200, Millipore), rabbit anti-Pdx1 (1∶200, Millipore), rabbit anti-Ptf1a (1∶600, a gift from Dr. Helena Edlund), rat anti-Somatostatin (1∶200, Chemicon), rabbit anti-Sox9 (1∶200, Chemicon), and chicken anti-YFP/GFP (1∶400, Abcam) followed by staining with Alexa Fluor tagged secondary antibodies (1∶500, Invitrogen) and mounting with DAPI-containing Vectashield media (Vector). For TUNEL analysis, ApopTag Plus Fluorescein In Situ Apoptosis Detection kit (Millipore) was used according to the manufacturer's protocol.

For embryo wholemount staining, tissues were processed as previously described [57] and stained with rat anti-E-Cadherin (1∶1,000, CalBiochem), followed by staining with Alexa Fluor 555 anti-rat secondary antibody (1∶500, Invitrogen).

For x-gal staining, tissues were fixed with 2% PFA and 0.25% Glutaraldehyde for 2 h and incubated overnight with 0.5 mg/ml x-gal solution (Roche), followed by a second round of fixation in 4% PFA overnight. Tissues were then embedded in paraffin, sectioned, and counter-stained with nuclear Fast Red (Vector).

For histological analysis, dissected tissues were fixed with Z-fix (Anatech), for 4 h, and embedded in paraffin wax. Tissue sections were stained with Meyer's Hematoxylin (Sigma) followed by staining with Eosin (Protocol).

Images were acquired using Zeiss ApoTome, Leica MZ FL3 and SP5, and Olympus IX70 microscopes.

### Quantifications

For all quantifications presented in this study, each transgenic tissue was processed and stained in parallel with a littermate control, with each analyzed group comprising at least three pairs of transgenic and control embryos (i.e., *n*≥3) as indicated in the figure legends. Throughout each analysis, images were acquired using the same exposure time and magnification. When MetaMorph software was used for image analysis, the same signal-to-noise threshold was applied throughout the experiment. For all measurements presented in this study, with the exception of the measurement of the mesenchymal area at e11.5 and Ptf1a^+^ cell numbers at e15.5, the following regimen was applied: the entire pancreatic tissue, including both dorsal and ventral buds, was embedded in paraffin wax and cut into 5 µm thick sections. Every fifth section (20% of total tissue) was then immuno-stained with indicated antibodies as described above. Images were acquired as detailed below and analyzed blindly.

For measurement of mesenchymal areas at e15.5 and e18.5, isolated pancreatic tissues from *Nkx3.2*-Cre;*R26*-YFP^flox^ embryos were stained with an anti-YFP antibody and a fluorescent secondary antibody and entire sections were automatically imaged using Olympus IX70 widefield microscope and MetaMorph software. Over-exposure of the tissue and DAPI staining were used to determine the edges of the section. Images were analyzed using MetaMorph software, which automatically measured the positive area in each channel. To determine the percentage of mesenchymal area, total YFP-positive area was divided by total tissue area of each section.

For β- and acinar cell mass, isolated e18.5 tissues (including both dorsal and ventral tissues) were dissected and weighed. Following fixation, tissues were embedded in paraffin wax, sectioned as described above, and immuno-stained with anti-Insulin and anti-Amylase antibodies. Images were acquired as described above for mesenchymal area measurement, and areas positive for either Amylase or Insulin, as well as the total pancreatic area, were automatically measured using MetaMorph software. To determine the fractions of the β- and acinar cell areas, total Insulin or Amylase positive area was divided by total tissue area. Cell mass was calculated as the fraction of Amylase^+^ or Insulin^+^ areas of the total pancreatic area multiplied by gross pancreas weight.

To calculate the β-cell/acinar cell ratio, for each embryo Insulin^+^ and Amylase^+^ area was determined as described above for cell mass measurement, and Insulin^+^ area was divided by Amylase^+^ area. For clarity, the ratio obtained in non-transgenic controls was set to “1.”

For quantification of Ngn3-expressing cells, whole e15.5 pancreatic tissues were isolated and processed as described above. Sections were stained with anti-Ngn3 antibody followed by fluorescent secondary antibody and images were then acquired as described above for mesenchymal area measurement, but positive cells were counted manually. To accommodate for potential differences in the developmental stage of the various litters analyzed, the number of transgenic Ngn3-positive cells was normalized to the number of Ngn3 cells counted in the corresponding non-transgenic littermate controls.

For cell proliferation, whole pancreatic tissue (including both dorsal and ventral tissues) was isolated from embryos e15.5 and older. From embryos at e13.5 or e14.5, pancreatic tissue was isolated together with the adjacent stomach and duodenum. Following fixation, tissues were paraffin-embedded and sectioned as described above. Tissue sections were stained with indicated antibodies and imaged using Zeiss ApoTome or Leica SP5 microscopes. For each section, the percentage of proliferating cells was determined via manual counting of either Ki67 or pHH3 positive cells divided by the number of total target cells.

To determine mesenchymal area at e11.5, *Nkx3*.2-Cre;*R26*-LacZ^f/+^ embryos were stained with X-gal as described above. Entire embryos were then cut to obtain 5 µm thick sections, and all sections were counterstained with FastRed dye and imaged using Zeiss ApoTome. The total dorsal pancreatic bud area, identified by its typical localization and morphology, and pancreatic mesenchyme area, identified by blue x-gal staining, were manually selected and measured using MetaMorph software.

For Ptf1a^+^ cell quantification, isolated e15.5 pancreatic tissue were fixed, embedded in OCT, frozen, and cryosectioned. 10 µm thick sections were used and every 10th section was stained (10% of total tissue). Whole sections were imaged using Leica SP5 confocal microscope and the number of positive cells was counted manually. To account for potential differences in developmental stage of each litter, the number of positive cells obtained for each transgenic animal was normalized to the number obtained from the non-transgenic littermate control.

### Statistics

*P* values were determined using unpaired, two-tailed student *t* test. Error bars in bar diagrams represent standard deviation of the samples.

## Supporting Information

Figure S1 *Nkx3.2*-Cre is not expressed by pancreatic neurons and endothelial cells. Analysis of p0 pancreatic tissues of *Nkx3.2*-Cre;*R26-YFP^f^* ^/+^ shows that YFP expressing cells do not express the neuronal marker Tuj1 or the endothelial marker PECAM1. (A) Immunofluorescence analysis for YFP (Green), PECAM1 (Red), and DAPI (Blue). (B) Flow cytometry analysis showing staining for PECAM1 of YFP-expressing (green histogram) and non-expressing cells (black line). For clarity, acinar (negative for YFP) and dead cells were excluded from the analysis based on size and DAPI staining, respectively. (C) Tissues were stained with antibodies against YFP (green) and Tuj1 (Red) and were counterstained with DAPI (blue).
(TIF)

Figure S2 In utero i.p. injection of Diphtheria Toxin (DT) to *Nkx3.2*-Cre;*DTR* embryos leads to death of pancreatic mesenchymal cell. (A–D) Graphic illustration of the injection procedure. A laparotomy was made (A) and the uterus, containing embryos, was delivered through the incision (B). Each embryo was injected with 5 µl of a solution containing varying concentrations of DT designed to result in a final concentration of 8 ng DT/gr embryo weight into the visible liver area (C). The uterus and embryos were placed back into the abdomen (D) and the incision was closed. Adapted from [35]. (E,F) Apoptotic pancreatic mesenchymal cell can be detected 4 h after DT injection to transgenic embryos. *Nkx3.2*-Cre;*DTR* embryos (F) and non-transgenic controls (E) injected with DT at e13.5 and analyzed 4 h after injection. Immunofluorescence staining for cleaved Caspase 3 (Green) as a marker for activation of the apoptotic machinery, for the epithelial marker E-Cadherin (Red) and for DAPI (blue) was performed. (G,H) Elimination of E-Cadherin-negative mesenchymal cells a day after DT injection. *Nkx3.2*-Cre;*DTR* (H) and non-transgenic embryos (G) were injected with DT at e13.5 and analyzed 24 h later for E-Cadherin (red) and DAPI (blue). (I,J) Elimination of DTR-expressing cells a day after DT injection. *Nkx3.2*-Cre;*DTR* were either injected with DT at e13.5 (J) or were left untreated (I). Tissues were harvested at e14.5 (24 h after DT injection) and stained for DTR (human hbEGF, red) and Pdx1 (green).
(TIF)

Figure S3 Gastrointestinal tract development is affected by mesenchymal ablation. Images of e18.5 stomach, pancreas, spleen, and gut of *Nkx3.2*-Cre;*DTR* embryos and non-transgenic controls injected with DT at e13.5.
(TIF)

Figure S4 Neurons and endothelial cells are present in mesenchyme-depleted pancreata. *Nkx3.2*-Cre;DTR and non-transgenic embryos were injected with DT at e13.5 and analyzed at e18.5. (A,B) Staining for the endothelial marker PECAM1 (red) indicates presence of blood vessels in transgenic pancreata (B). (C,D) Whole mount staining against the endothelial marker PECAM1 (brown) was performed. Images reveal dense vasculature in DT-treated transgenic pancreata (D) and control (C). (C′,D′) A higher magnification of the areas marked with a white box in (C) and (D), respectively. (E,F) Staining for the neuronal marker Tuj1 (green) indicates presence of neurons in transgenic pancreata (F), similar to non-transgenic control (F).
(TIF)

Figure S5 β-catenin is not localized to the membrane of pancreatic mesenchymal cells, and its elimination in the mesenchyme does not affect epithelial growth prior to the onset of mesenchymal Wnt signaling. (A) β-catenin is localized to the membrane of Pdx1^+^ epithelial cells but not to the membrane of Nkx3.2/YFP^+^ mesenchymal cells. *Nkx3.2*-Cre;*R26*-YFP^f/+^ e14.5 pancreatic tissue was stained for YFP (green), β-catenin (red), and Pdx1 (blue). Left panel shows all three markers, while middle and right panels show only indicated markers. (B) Normal epithelial size in *Nkx3.2*-Cre;*βcat* ^f/f^ at e12.5. Embryos were stained with H&E and epithelial area was measured and compared to non-transgenic littermates (which was set to “1”). Epithelial area in mutant embryos (black bar) was comparable to that of non-transgenic animals (non-tg, gray bar, set to “1”). *n* = 3.
(TIF)

Text S1 Supporting materials and methods. The procedures and reagents used to generate the data presented in the supporting figures are described in detail.
(DOC)

## Abbreviations

DT: Diphtheria Toxin
DTA: Diphtheria Toxin active A subunit
DTR: Diphtheria Toxin receptor
hbEGF: heparin binding epidermal growth factor
hESC: human embryonic stem cell
Ngn3: Neurogenin 3
RA: Retinoic Acid
