# Supplementary figures and images for: Pancreatic Mesenchyme Regulates Epithelial Organogenesis throughout Development
Source: PLoS Biol. 2011 Sep 6;9(9):e1001143. doi: 10.1371/journal.pbio.1001143 (PMC3167782; doi:10.1371/journal.pbio.1001143)

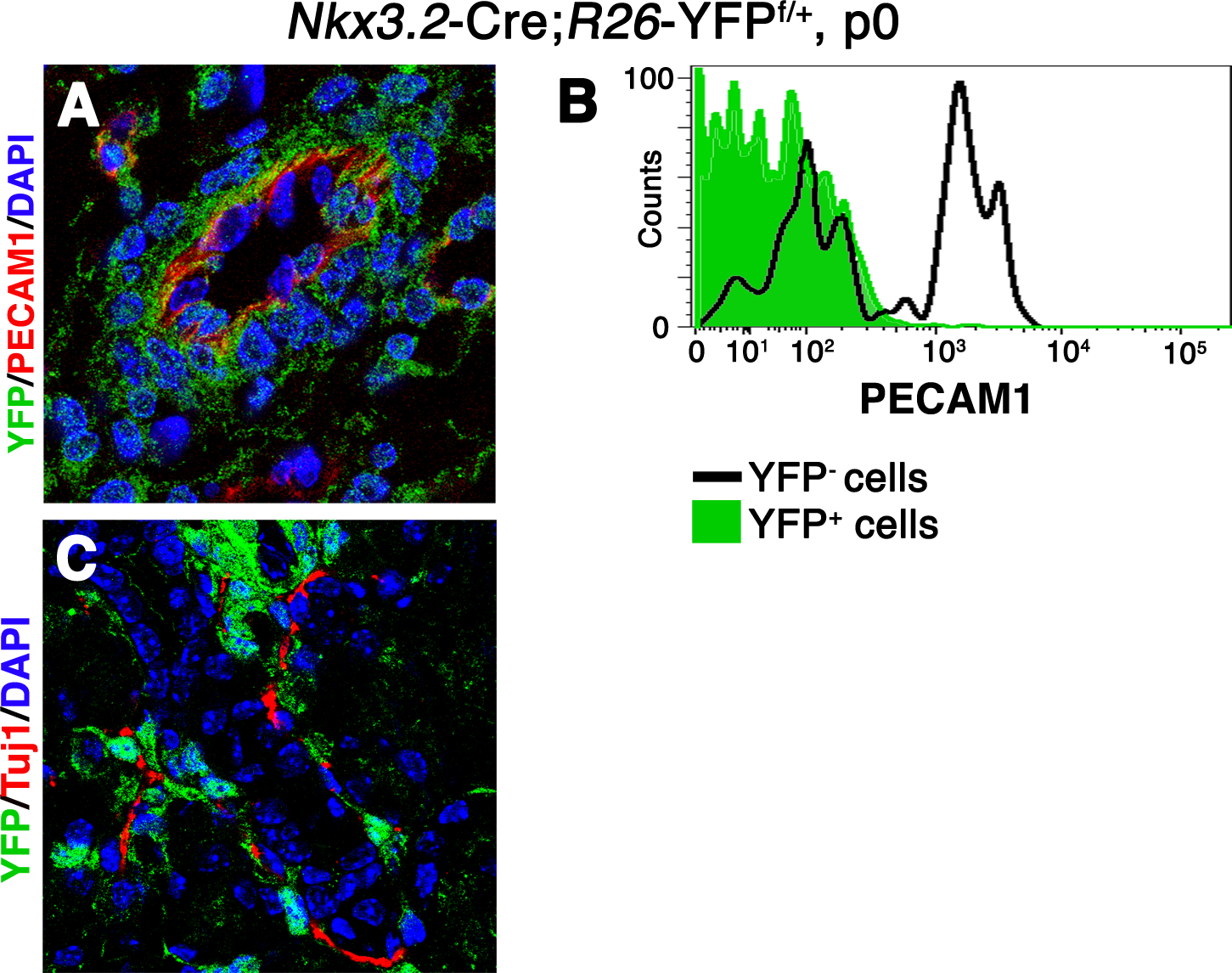

Supplement: Figure S1 — Nkx3.2-Cre is not expressed by pancreatic neurons and endothelial cells. Analysis of p0 pancreatic tissues of Nkx3.2-Cre;R26-YFPf /+ shows that YFP expressing cells do not express the neuronal marker Tuj1 or the endothelial marker PECAM1. (A) Immunofluorescence analysis for YFP (Green), PECAM1 (Red), and DAPI (Blue). (B) Flow cytometry analysis showing staining for PECAM1 of YFP-expressing (green histogram) and non-expressing cells (black line). For clarity, acinar (negative for YFP) and dead cells were excluded from the analysis based on size and DAPI staining, respectively. (C) Tissues were stained with antibodies against YFP (green) and Tuj1 (Red) and were counterstained with DAPI (blue). (TIF) [file pbio.1001143.s001.tif]

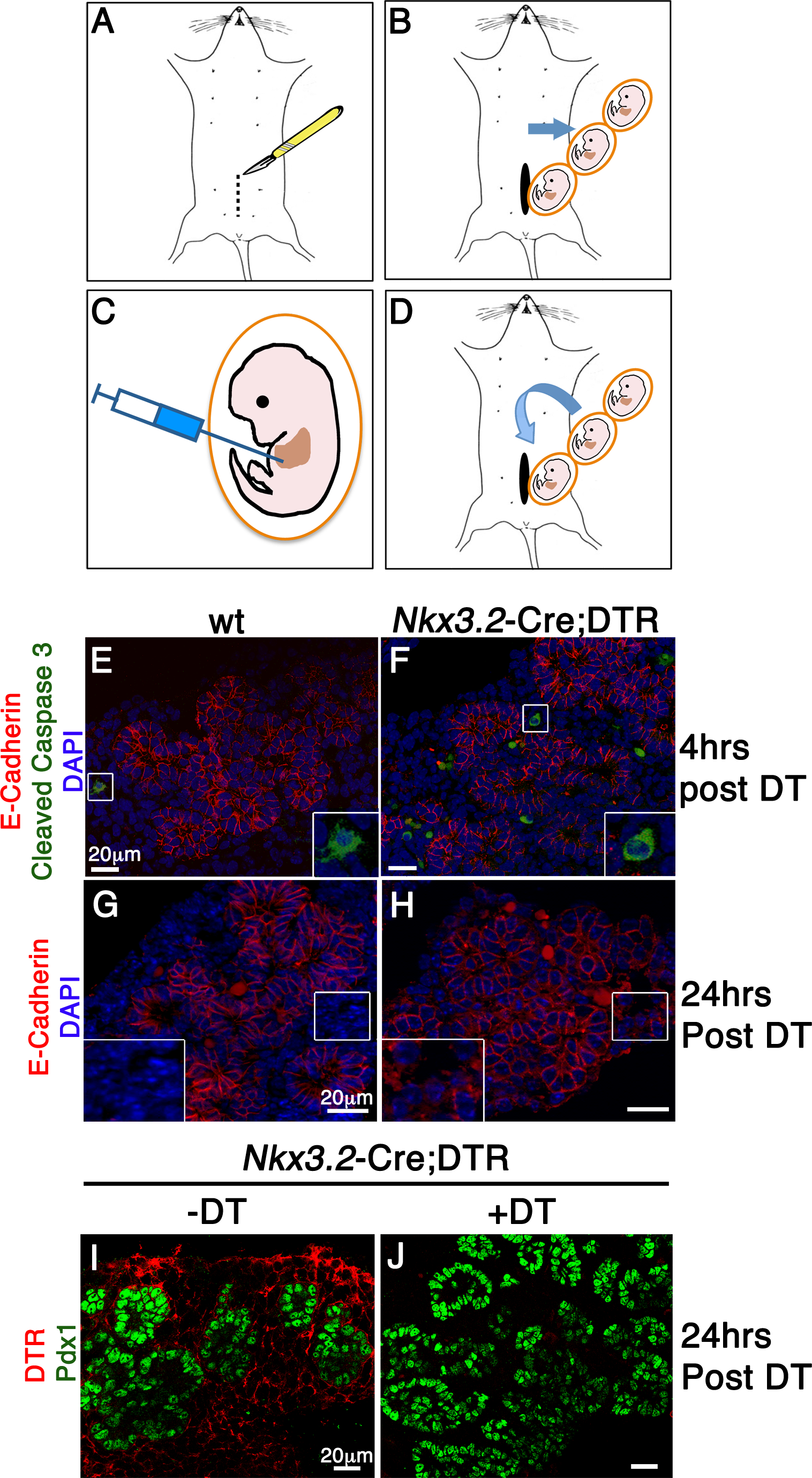

Supplement: Figure S2 — In utero i.p. injection of Diphtheria Toxin (DT) to Nkx3.2-Cre;DTR embryos leads to death of pancreatic mesenchymal cell. (A–D) Graphic illustration of the injection procedure. A laparotomy was made (A) and the uterus, containing embryos, was delivered through the incision (B). Each embryo was injected with 5 µl of a solution containing varying concentrations of DT designed to result in a final concentration of 8 ng DT/gr embryo weight into the visible liver area (C). The uterus and embryos were placed back into the abdomen (D) and the incision was closed. Adapted from [35]. (E,F) Apoptotic pancreatic mesenchymal cell can be detected 4 h after DT injection to transgenic embryos. Nkx3.2-Cre;DTR embryos (F) and non-transgenic controls (E) injected with DT at e13.5 and analyzed 4 h after injection. Immunofluorescence staining for cleaved Caspase 3 (Green) as a marker for activation of the apoptotic machinery, for the epithelial marker E-Cadherin (Red) and for DAPI (blue) was performed. (G,H) Elimination of E-Cadherin-negative mesenchymal cells a day after DT injection. Nkx3.2-Cre;DTR (H) and non-transgenic embryos (G) were injected with DT at e13.5 and analyzed 24 h later for E-Cadherin (red) and DAPI (blue). (I,J) Elimination of DTR-expressing cells a day after DT injection. Nkx3.2-Cre;DTR were either injected with DT at e13.5 (J) or were left untreated (I). Tissues were harvested at e14.5 (24 h after DT injection) and stained for DTR (human hbEGF, red) and Pdx1 (green). (TIF) [file pbio.1001143.s002.tif]

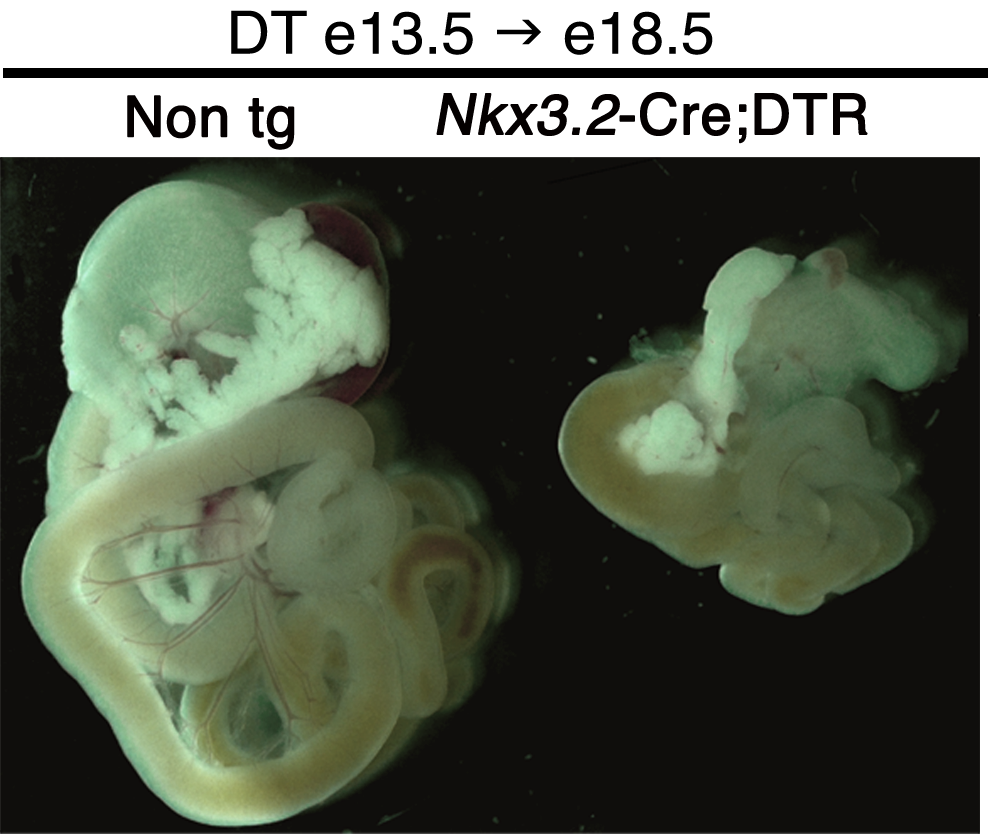

Supplement: Figure S3 — Gastrointestinal tract development is affected by mesenchymal ablation. Images of e18.5 stomach, pancreas, spleen, and gut of Nkx3.2-Cre;DTR embryos and non-transgenic controls injected with DT at e13.5. (TIF) [file pbio.1001143.s003.tif]

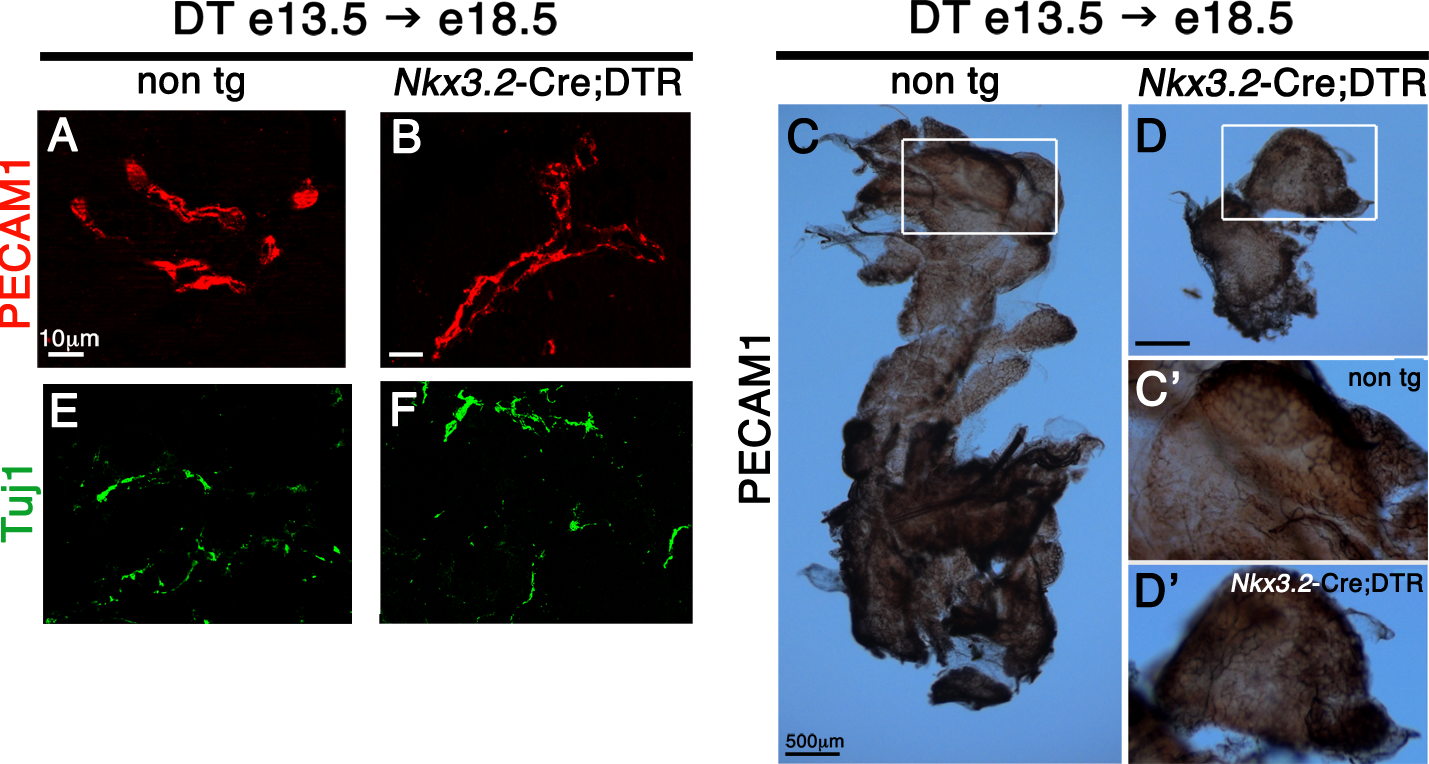

Supplement: Figure S4 — Neurons and endothelial cells are present in mesenchyme-depleted pancreata. Nkx3.2-Cre;DTR and non-transgenic embryos were injected with DT at e13.5 and analyzed at e18.5. (A,B) Staining for the endothelial marker PECAM1 (red) indicates presence of blood vessels in transgenic pancreata (B). (C,D) Whole mount staining against the endothelial marker PECAM1 (brown) was performed. Images reveal dense vasculature in DT-treated transgenic pancreata (D) and control (C). (C′,D′) A higher magnification of the areas marked with a white box in (C) and (D), respectively. (E,F) Staining for the neuronal marker Tuj1 (green) indicates presence of neurons in transgenic pancreata (F), similar to non-transgenic control (F). (TIF) [file pbio.1001143.s004.tif]

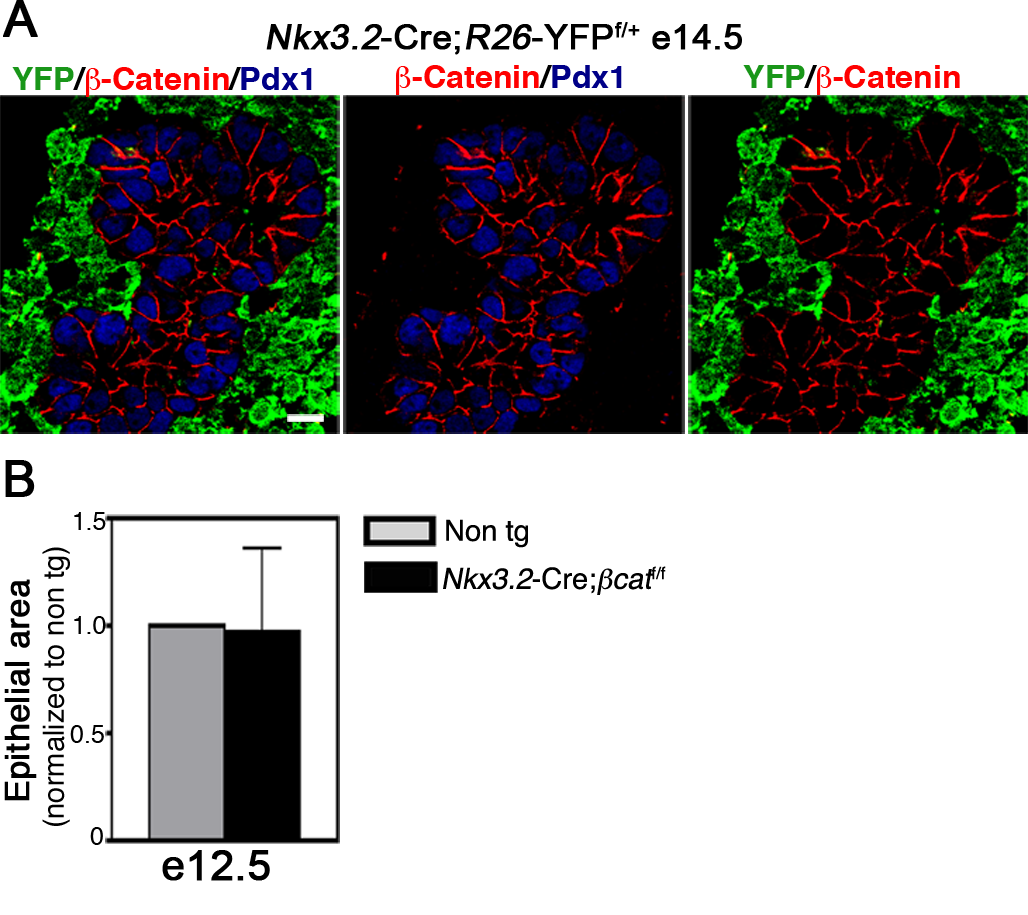

Supplement: Figure S5 — β-catenin is not localized to the membrane of pancreatic mesenchymal cells, and its elimination in the mesenchyme does not affect epithelial growth prior to the onset of mesenchymal Wnt signaling. (A) β-catenin is localized to the membrane of Pdx1+ epithelial cells but not to the membrane of Nkx3.2/YFP+ mesenchymal cells. Nkx3.2-Cre;R26-YFPf/+ e14.5 pancreatic tissue was stained for YFP (green), β-catenin (red), and Pdx1 (blue). Left panel shows all three markers, while middle and right panels show only indicated markers. (B) Normal epithelial size in Nkx3.2-Cre;βcat f/f at e12.5. Embryos were stained with H&E and epithelial area was measured and compared to non-transgenic littermates (which was set to “1”). Epithelial area in mutant embryos (black bar) was comparable to that of non-transgenic animals (non-tg, gray bar, set to “1”). n = 3. (TIF) [file pbio.1001143.s005.tif]
