# Supplementary material for: Pancreatic Mesenchyme Regulates Epithelial Organogenesis throughout Development
Source: PLoS Biol. 2011 Sep 6;9(9):e1001143. doi: 10.1371/journal.pbio.1001143 (PMC3167782; doi:10.1371/journal.pbio.1001143)
Supplement: Text S1 — Supporting materials and methods. The procedures and reagents used to generate the data presented in the supporting figures are described in detail. (DOC) [file pbio.1001143.s006.doc]

**Text S1**

**Immunostaining**

Following dissection, embryos were fixed with Z-fix (Anatech), embedded in paraffin wax, sectioned and stained with mouse anti-βcatenin (1:200, BD), rabbit anti-cleaved Caspase 3 (1:200, Cell Signaling), mouse anti-E-Cadherin (1:200, BD), mouse anti-human hbEGF (DTR, 1:200, NBL), rabbit anti-Pdx1 (1:200, Millipore) rabbit anti-Tuj1 (βIII Tubulin, 1:200, Sigma) and chicken anti-YFP/GFP (1:400, Abcam) followed by Alexa Fluor tagged secondary antibodies (1:500, Invitrogen).

For PECAM1 immunofluorescence, 4ng/ml proteinase K was used for antigen retrieval prior to staining with rat anti-PECAM1/CD31 (1:200, BD) as described [2], followed by staining with AlexaFluor 555 secondary antibody (1:500, Invitrogen). For pancreas whole mount PECAM1 staining, tissues were processed as described [3].

**Flow Cytometry**

Pancreata were digested for with 2.5 mg/ml Collagenase D (Roche) followed by incubation with 0.05% Trypsin and then with 2U/ml Dispase (Invitrogen), all at 37^0^C. Cells were stained with PE conjugated anti PECAM1/CD31 antibody (1:100, BD) and analyzed using SLII (BD).

**Quantifications**

For epithelial area measurement, whole e12.5 embryos were fixed, embedded in paraffin and cut into 5μm sections. All sections containing pancreatic tissue were stained with Hematoxylin (sigma) and Eosin (Protocol) and imaged using a Zeiss ApoTome microscope. Pancreatic epithelium was identified according to typical morphology and its area was selected manually and measured using MetaMorph software.

**References**

1. Nijagal A, Le T, Wegorzewska M, Mackenzie TC (2011) A mouse model of in utero transplantation. J Vis Exp.

2. Pierreux CE, Cordi S, Hick AC, Achouri Y, Ruiz de Almodovar C, et al. (2010) Epithelial: Endothelial cross-talk regulates exocrine differentiation in developing pancreas. Dev Biol 347: 216-227.

3. Villasenor A, Chong DC, Henkemeyer M, Cleaver O (2010) Epithelial dynamics of pancreatic branching morphogenesis. Development 137: 4295-4305.
